# Cannabinoid CB1 Receptor in Nociceptors Mediates Postoperative Analgesia via ASIC3 Inhibition

**DOI:** 10.1002/advs.77925

**Published:** 2026-09-27

**Authors:** Kangtai Xu, Luyao Ji, Haoyi Yang, Xiying Chen, Qiaodan Hu, Haimei He, Jiawei Wu, Leyan Shan, Shihui Feng, Yingzhi Li, Xiongchang Tan, Hao Wang, Zilong Wang, Chaoran Wu

**Affiliations:** ^1^ Department of Anesthesiology The Second Clinical Medical College Shenzhen People's Hospital Jinan University Shenzhen Guangdong China; ^2^ Department of Anesthesiology and Clinical Research Institute Shantou Clinical Medical College The First Affiliated Hospital Jinan University Guangzhou China; ^3^ Department of Medical Neuroscience School of Medicine Southern University of Science and Technology Shenzhen Guangdong China; ^4^ Key University Laboratory of Metabolism and Health of Guangdong School of Medicine Southern University of Science and Technology Shenzhen Guangdong China; ^5^ SUSTech Homeostatic Medicine Institute School of Medicine Southern University of Science and Technology Shenzhen Guangdong China

**Keywords:** ASIC3, CB_1_R, peripheral analgesia, postoperative pain, sensory nerve

## Abstract

Peripherally restricted analgesics devoid of central side effects are urgently needed for postoperative pain management. The cannabinoid type‐1 receptor (CB_1_R) expressed on primary sensory neurons represents an attractive peripheral target, yet its specific role and mechanism in postoperative pain remain poorly defined. Here, by constructing a mouse model of plantar incision and combining with nociceptor‐selective *Cnr1* knockout and site‐specific pharmacology, we revealed that activation of CB_1_R in primary sensory neurons robustly alleviates postoperative mechanical allodynia. Peripheral CB_1_R co‐localizes and physically associates with the proton‐sensing ion channel ASIC3 in CGRP^+^ peptidergic nociceptors in the dorsal root ganglion (DRG). The activation of CB_1_R signaling suppresses ASIC3‐mediated inward currents and subsequent calcium transients, thereby reducing neuronal excitability and ERK_1/2_ phosphorylation in nociceptors. Consequently, targeted activation of CB_1_R or blockade of ASIC3 signaling significantly alleviated postoperative pain hypersensitivity and promoted pain resolution. This study elucidated a novel peripheral mechanism by which CB_1_R‐ASIC3 physical and functional coupling in peptidergic nociceptors drives the resolution of incisional pain and provided a theoretical basis for the development of peripherally acting, non‐opioid analgesic strategies.

## Graphical abstract

CB_1_R and ASIC3 are co‐expressed in CGRP^+^ nociceptors of mouse DRG. Plantar incision triggers an upregulation of ASIC3 and activation of ASIC3^+^ neurons, driving mechanical allodynia. CB_1_R activation suppresses ASIC3 function and downstream ERK_1/2_ phosphorylation, thereby braking nociceptive signaling and accelerating pain resolution. This peripheral CB_1_R‐ASIC3 axis constitutes an endogenous analgesic mechanism and a promising therapeutic target for postoperative pain.

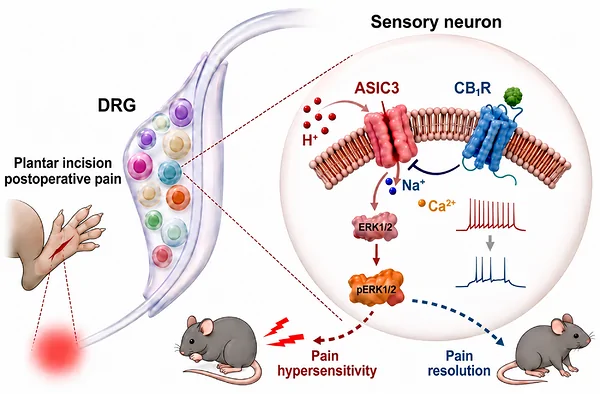

## 1 Introduction

Postoperative pain remains a significant clinical burden, with up to 75% of surgical patients experiencing moderate‐to‐severe pain despite advances in surgical and analgesic techniques [1]. Surgical tissue injury creates an acidic and pro‐inflammatory milieu, where protons and inflammatory mediators not only activate but also sensitize peripheral nociceptors, thereby driving pain hypersensitivity [2]. Inadequate pain management, particularly the heavy reliance on opioids, remains common and significantly impairs patient recovery, exacerbates neuroinflammatory responses to the central nervous system, and increases the risk of chronic pain transition [3]. Consequently, elucidation of the pathological mechanisms that underlie postoperative pain and the development of targeted nonopioid therapeutic strategies are urgently needed.

The cannabinoid type‐1 receptor (CB_1_R), one of the most abundant G protein‐coupled receptors in the nervous system, is a compelling target for pain modulation [4]. However, the therapeutic application of centrally active cannabinoids has been limited by their psychoactive and other cannabimimetic side effects [5, 6, 7]. Previous studies have established that endogenous cannabinoid signaling through CB_1_R and CB_2_R contributes to the resolution of postoperative pain by limiting spinal neuroinflammation and astrocytic activation in rats [8]. More recently, the analgesic effect of acetaminophen in incisional pain has been shown to require CB_1_R activation, likely at a central site of action [9]. However, whether selective activation of peripheral CB_1_R on primary sensory neurons is sufficient to produce postoperative analgesia, and the molecular mechanisms by which such peripherally restricted analgesia might be achieved, remain largely unexplored. Notably, transcriptomic profiling has shown that surgical incision induces pain‐related gene expression changes predominantly in skin, muscle, and dorsal root ganglia (DRG), with minimal alterations in spinal cord and brain [10]. This pattern suggests that acute postoperative pain is driven primarily by peripheral nociceptor sensitization, rather than by the central sensitization mechanisms that dominate chronic pain states. As the first and obligatory relay station for sensory input, the regulation and processing of pain signals in the DRG hold enormous potential for the treatment of postoperative pain. Therefore, we hypothesized that selectively targeting peripheral CB_1_R in DRG neurons could alleviate acute postoperative pain without central adverse effects.

Tissue acidosis is a hallmark of surgical injury, and the proton‐gated acid‐sensing ion channel 3 (ASIC3), a sodium channel highly expressed in peripheral sensory neurons, is a key sensor of acidosis in peripheral nociceptors, contributing to injury‐ and inflammation‐induced pain [11, 12]. Importantly, Deval et al. demonstrated that ASIC3 is upregulated in DRG neurons after plantar incision and that pharmacological blockade or siRNA‐mediated knockdown of ASIC3 significantly attenuates postoperative pain behaviors in rats [13]. CB_1_R was initially characterized as a GPCR that couples primarily to pertussis toxin‐sensitive G_i/o_ signal transduction proteins and, when activated, inhibits adenylate cyclase to reduce intracellular cyclic AMP (cAMP) levels and modulate downstream cascades under the control of protein kinase A (PKA) [14, 15]. Previous studies have suggested that, in addition to this canonical signaling, CB_1_R can modulate non‐selective cation channels such as hyperpolarization‐activated cyclic nucleotide‐gated (HCN) channels and P2X3 receptor [16, 17, 18]. However, whether CB_1_R signaling engages ASIC3 in the context of postoperative pain remains uninvestigated.

In the present study, we established a model of postoperative pain caused by plantar incision and explored the role of peripheral CB_1_R in incision‐induced postoperative pain and its interactions with the ASIC3. We demonstrated that CB_1_R and ASIC3 co‐localize and physically associate in CGRP^+^ peptidergic nociceptors, and that CB_1_R agonism suppresses ASIC3‐mediated inward currents, calcium transients, and downstream ERK_1/2_ phosphorylation. This further inhibits the sensitization of sensory neurons, thereby mediating postoperative analgesia. These findings highlight the peripheral CB_1_R‐ASIC3 axis as a promising therapeutic target for developing non‐opioid, peripherally restricted analgesics with improved safety profiles in postoperative pain management.

## 2 Results

### 2.1 Peripheral CB_1_R Activation Alleviates Incision‐Induced Postoperative Pain

**FIGURE 1:**
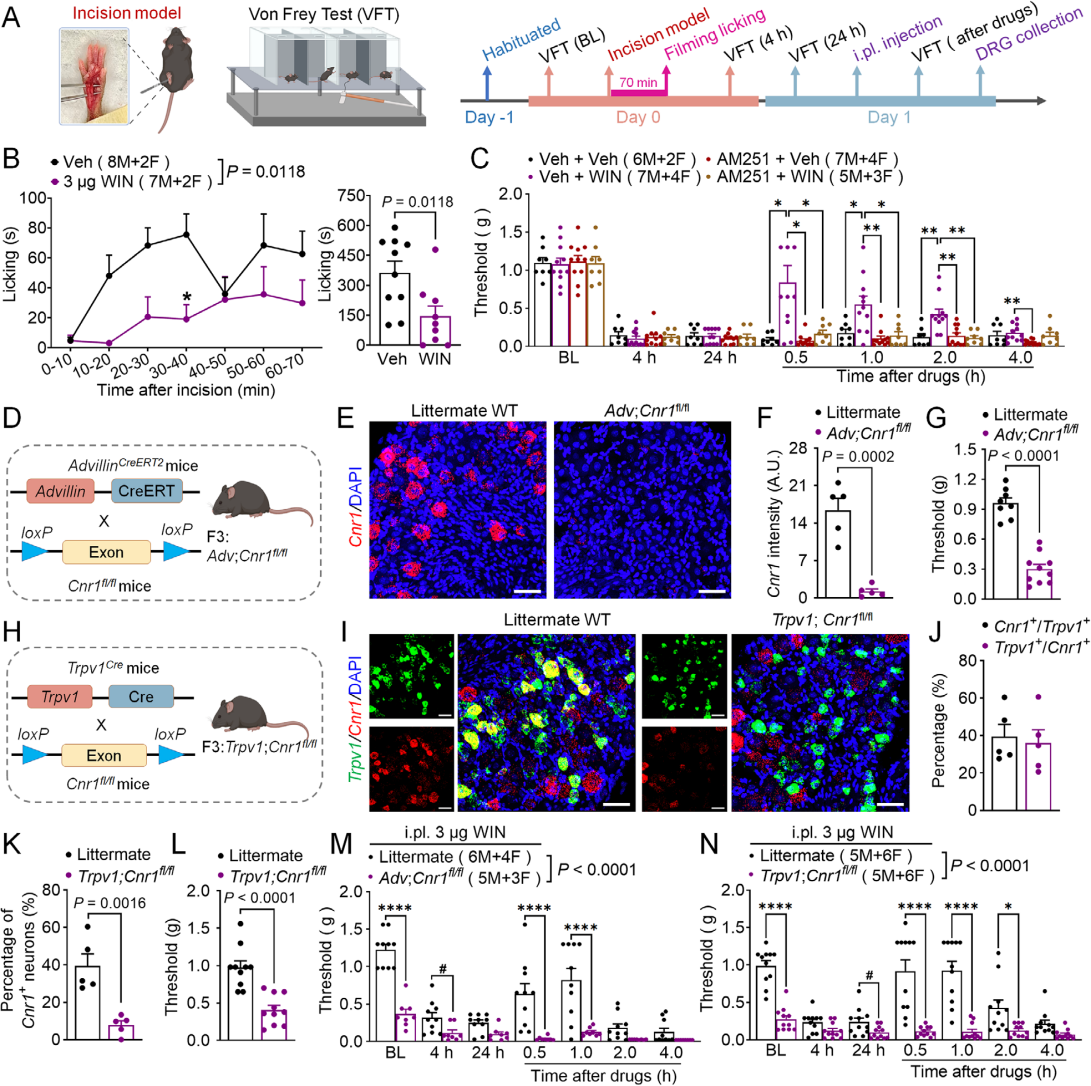
Peripheral CB_1_R activation alleviates incision‐induced postoperative pain. (A) Schematic of the plantar incision model and behavioral testing timeline. (B) Intraplantar (i.pl.) injection of WIN 55,212‐2 (WIN) immediately after plantar incision suppresses incision‐evoked licking behavior. Left: time course; Two‐way ANOVA with Sidak correction for multiple comparisons, ^*^ *p* < 0.05. Right: total licking duration; Unpaired t tests; n = 9–10 mice/group. (C) WIN‐induced reversal of mechanical allodynia on postoperative day 1 is fully blocked by co‐administration of the CB_1_R antagonist AM251. Two‐way ANOVA with Sidak correction for multiple comparisons; n = 8–11 mice/group. (D) Strategy of constructing *Adv*;*Cnr1* ^fl/fl^ mice. (E,F) Representative RNAscope images and quantification of *Cnr1* mRNA in DRG of *Adv*;*Cnr1* ^fl/fl^ mice. Scale bar = 50 µm, 20× objective, zoom = 1.28×. Unpaired t tests; n = 5 mice/group, 4–5 sections/mouse. (G) Baseline mechanical paw withdrawal thresholds (PWT) in *Adv*;*Cnr1* ^fl/fl^ mice. Unpaired t tests; n = 8–10 mice/group. (H) Strategy of constructing *Trpv*1;*Cnr1* ^fl/fl^ mice. (I–K) RNAscope analysis and quantification of *Cnr1* and *Trpv1* in DRG of *Trpv*1;*Cnr1* ^fl/fl^ mice. Scale bar = 50 µm, 20 × objective, zoom = 1.28 ×. Unpaired t tests; n = 5 mice/group, 4–5 sections/mouse. (L) Baseline PWT in *Trpv1*;*Cnr1* ^fl/fl^ mice. Unpaired t tests; n = 10 mice/group. (M) PWT of *Adv*;*Cnr1* ^fl/fl^ mice after i.pl. WIN on day 1 after the incision. (N) PWT in *Trpv1*;*Cnr1* ^fl/fl^ mice after i.pl. WIN on day 1 post‐incision. Two‐way ANOVA with Sidak correction for multiple comparisons. ^*^ *p* < 0.05, ^**^ *p* < 0.01, and ^****^ *p* < 0.0001; **^#^** *p* < 0.05 (post‐drug time points were excluded during the analysis); n = 8–11 mice/group, means ± SEM. M, male; F, female; BL, baseline; Veh, vehicle; WIN, WIN 55,212‐2.

To define the contribution of peripheral CB_1_R to postoperative pain, we employed the plantar incision model (Figure 1A). Intraplantar (i.pl.) administration of the synthetic cannabinoid agonist WIN 55 212‐2 (WIN; 3 µg) significantly suppressed incision‐evoked licking behavior that occurred immediately after the incision (Figure 1B), and reversed mechanical allodynia in both male and female wild‐type (WT) mice on postoperative day 1 (Figure S1A,B). We also evaluated the analgesic effects of contralateral intraplantar and dorsal subcutaneous (s.c.) injection of the same dose of WIN. At 24 h after plantar incision, compared to intraplantar injection of WIN into the ipsilateral (incised), injection of the same dose of WIN into the contralateral (non‐incised) paw and dorsal subcutaneous had no significant effects on mechanical allodynia in the incised paw (Figure S1C). These findings demonstrated that the analgesic effect of intraplantar WIN is restricted to the peripheral injection site. Additionally, gait analysis found that mice exhibited a significant reduction in both stance time and the contact area of the ipsilateral hindpaw at 24 h after plantar incision (Figure S1D–I). Intraplantar injection of WIN significantly reversed these deficits, restoring both paw stance time and contact area toward pre‐injury levels. Critically, the results of the rotarod test and the open field test indicated that in naive mice, intraplantar injection of WIN did not affect the mice's motor coordination and spontaneous locomotor activity (Figure S1J–M). These data demonstrated that peripheral CB_1_R activation not only attenuates evoked mechanical allodynia but also improves spontaneous, functional measures of pain‐related behavior. Notably, mechanical allodynia assessment demonstrated that this antinociceptive effect was fully blocked by pre‐administration of the selective CB_1_R antagonist AM251 (Figure 1C). However, the analgesic efficacy of WIN was fully preserved in CB_2_R global‐knockout (CB_2_R^−/−^) mice (Figure S1N), and the different doses of the CB_2_R‐selective agonist AM1241 failed to attenuate incision‐induced mechanical allodynia in WT mice (Figure S1O). These results demonstrate that peripheral WIN produces postoperative analgesia specifically through CB_1_R, with negligible contribution from CB_2_R.

To genetically validate these findings, we generated *Advillin^CreERT2^*;*Cnr1^flox/flox^* (*Adv;Cnr1^fl/fl^*) mice, in which tamoxifen‐inducible recombination depletes *Cnr1* in almost all sensory neurons (Figure 1D and Figure S2A). RNAscope in situ hybridization confirmed a marked reduction of *Cnr1* mRNA in DRG neurons from *Adv;Cnr1^fl/fl^* mice (Figure 1E,F). Notably, *Adv;Cnr1^fl/fl^* mice exhibited significant mechanical hypersensitivity under naïve conditions (Figure 1G). To further evaluate the specific nociceptor population, we generated *Trpv1;Cnr1^fl/fl^* mice (Figure 1H and Figure S2B). The RNAscope results indicated that approximately 40% of *Cnr1* is expressed in TRPV1‐expressing neurons in the DRG of WT mice, and which was selectively deleted in TRPV1 neurons of *Trpv1;Cnr1^fl/fl^* mice (Figure 1I–K). These mice similarly displayed elevated basal mechanical sensitivity (Figure 1L).

We next assessed the functional requirement for CB_1_R expressed in sensory neurons in cannabinoid‐mediated postoperative analgesia. The anti‐allodynic effect of i.pl. WIN was completely lost in both *Adv;Cnr1^fl/fl^* mice and *Trpv1;Cnr1^fl/fl^* mice (Figure 1M,N). Consistent with a tonic inhibitory role of peripheral CB_1_R, conditional *Cnr1* deletion exacerbated incision‐induced mechanical allodynia (Figure S2C,D), and i.pl. injection of AM251 alone was sufficient to provoke mechanical hypersensitivity in WT mice (Figure S2E). Furthermore, we assessed thermal nociception. In contrast to the profound mechanical hypersensitivity, both *Adv;Cnr1^fl/fl^* mice and *Trpv1;Cnr1^fl/fl^* mice exhibited normal latencies in the hot‐plate and tail‐flick tests (Figure S3A–F). This demonstrates that endogenous CB_1_R signaling in primary sensory neurons specifically gates physiological tactile sensitivity and postoperative mechanical allodynia, without modulating acute thermal nociception.

### 2.2 ASIC3‐Expressing Nociceptors are Activated in the DRG and Contribute To Postoperative Nociception

**FIGURE 2:**
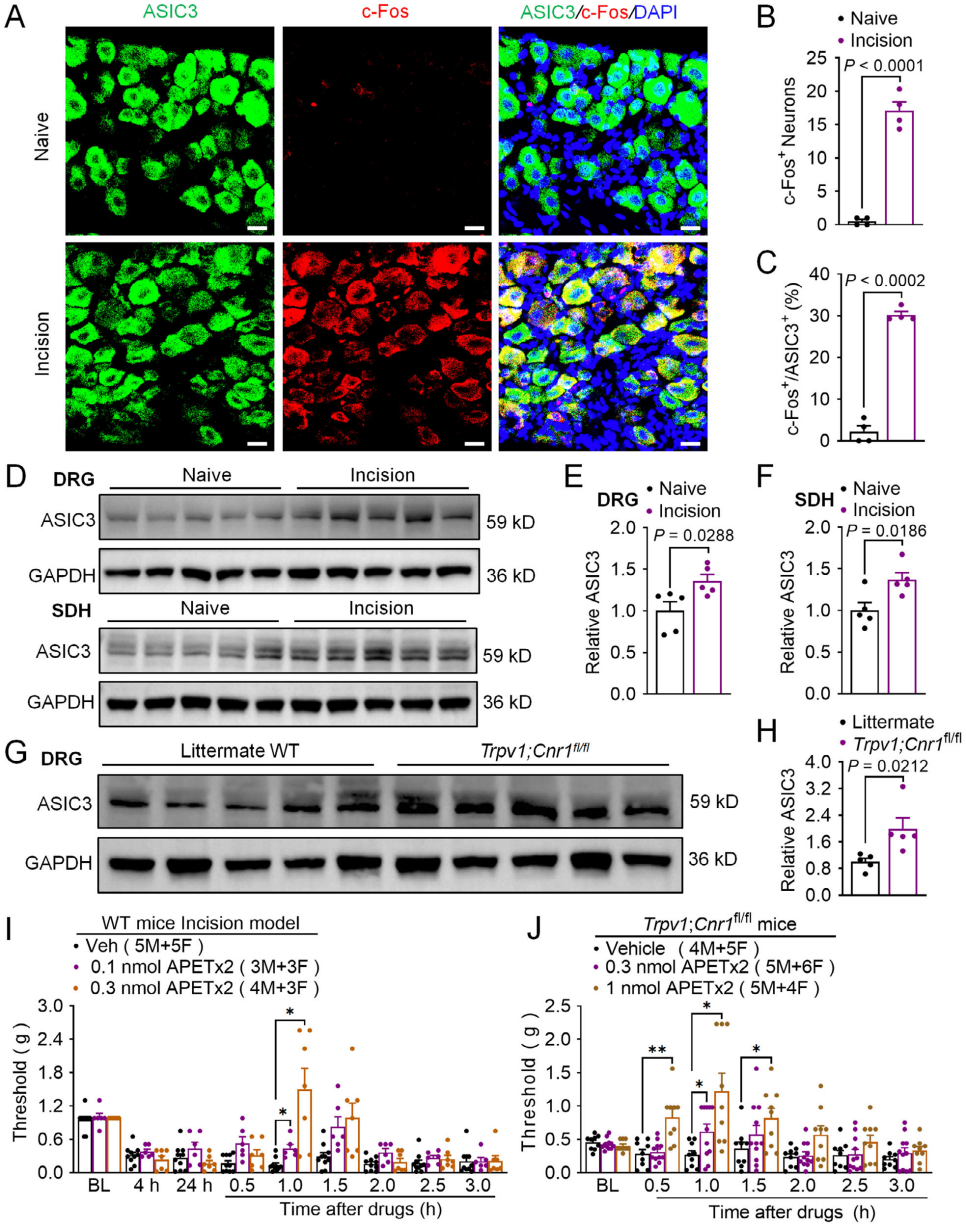
ASIC3‐expressing nociceptors are activated in the DRG and contribute to incision‐induced postoperative pain. (A–C) Representative c‐Fos immunostaining images and quantification in WT DRG sections on postoperative day 1. Scale bar = 20 µm, 20 × objective, zoom = 1.97 ×. Unpaired t tests; n = 4 mice/group, 3–4 sections/mouse. (D–F) Western blot analysis of ASIC3 expression in WT mouse DRG and spinal dorsal horn (SDH) on postoperative day 1. Unpaired t tests; n = 5 mice/group. (G,H) ASIC3 protein levels in the DRG of littermate WT mice and *Trpv1*;*Cnr1* ^fl/fl^ mice on day 1 post‐incision. Unpaired t tests; n = 5 mice/group. (I) i.pl. injection of the selective ASIC3 blocker APETx2 on postoperative day 1 robustly reverses mechanical allodynia in WT mice. (J) PWT following i.pl. injection of the ASIC3 blocker APETx2 in naïve *Trpv1*;*Cnr1* ^fl/fl^ mice. Two‐way ANOVA with Sidak's test for multiple comparisons; ^*^ *p* < 0.05, ^**^ *p* < 0.01. n = 6–11 mice/group; means ± SEM. M, male; F, female; BL, baseline; Veh, vehicle.

Surgical incision creates a profoundly acidic tissue environment, and ASIC3 as one of the acid‐sensing ion channels is widely recognized as a key proton sensor in nociceptors that drives pain associated with tissue injury and inflammation [13, 19, 20, 21]. We therefore reasoned that ASIC3 may represent a functionally relevant downstream target of peripheral CB_1_R signaling in the context of incisional pain. To test this hypothesis, we first confirmed whether ASIC3‐expressing neurons are engaged during postoperative pain in mice. In the DRG of mice subjected to plantar incision, c‐Fos immunostaining reveals that approximately 30% of ASIC3^+^ neurons were activated (Figure 2A–C). Concurrently, ASIC3 protein levels were markedly upregulated in both DRG and spinal cord dorsal horns (SDH) (Figure 2D–F) in postoperative pain, consistent with enhanced nociceptive signaling. Additionally, in the incision model, ASIC3 expression had a further increase in the DRG of *Trpv1;Cnr1^fl/fl^* mice than in littermate WT mice (Figure 2G,H). Notably, basal ASIC3 protein expression in the DRG is significantly elevated in *Adv;Cnr1^fl/fl^* mice and *Trpv1;Cnr1^fl/fl^* mice compared with WT controls under naïve conditions (Figure S4A–D). This finding indicates that CB_1_R tonically suppresses ASIC3 expression and that genetic deletion of CB_1_R leads to a compensatory upregulation that may contribute to the mechanical hypersensitivity observed in these animals.

We next assessed the functional contribution of ASIC3 to postoperative pain. Intraplantar injection of the selective ASIC3 blocker APETx2 on postoperative day 1 significantly reverses mechanical allodynia in WT mice (Figure 2I), directly implicating ASIC3 activity in the maintenance of incision‐induced hypersensitivity. Furthermore, in naïve *Trpv1;Cnr1^fl/fl^* mice, which exhibit pronounced basal mechanical allodynia, APETx2 significantly alleviates this hypersensitivity (Figure 2J). This result confirms that ASIC3 contributes to the pain phenotype caused by CB_1_R deletion. Together, these results establish that ASIC3 activation and upregulation in sensory neurons are critical for the development of both incision‐induced and CB_1_R‐deficiency‐induced mechanical allodynia.

### 2.3 CB_1_R Activation Suppresses ASIC3‐Mediated Nociceptive Behavior

**FIGURE 3:**
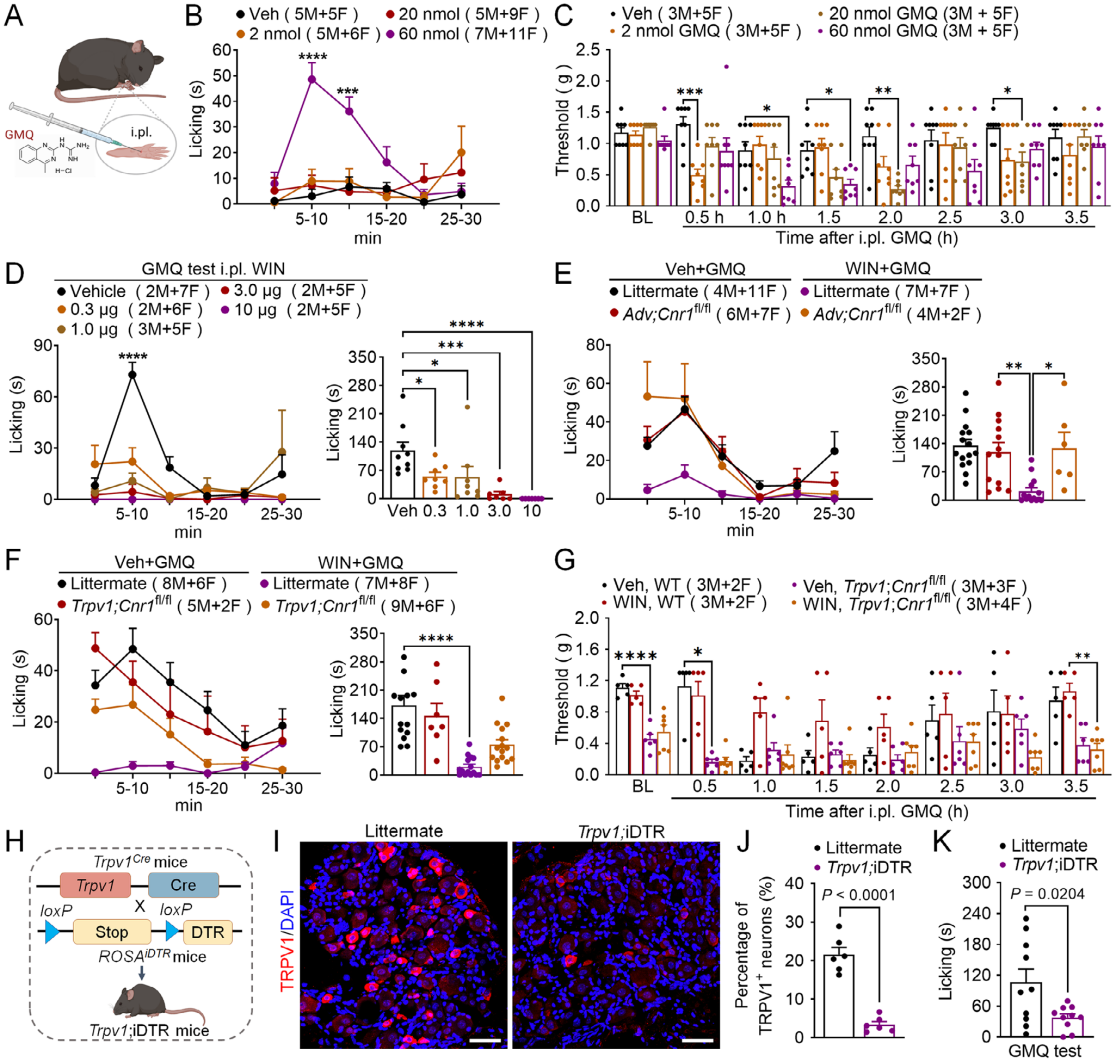
The activation of CB_1_Rs inhibits ASIC3‐mediated nociceptive behavior. (A) Schematic of the GMQ test for assessing ASIC3‐driven nociception. (B,C) i.pl. GMQ evokes robust licking behavior and mechanical allodynia in WT mice. (D) Pretreatment with WIN (i.pl.) 15 min dose‐dependent inhibits 60 nmol GMQ‐induced licking behaviors in WT mice (left: time course; right: total licking time). (E,F) Pretreatment with WIN (i.pl.) fails to suppress GMQ‐induced licking in *Adv*;*Cnr1* ^fl/fl^ mice and *Trpv1*;*Cnr1* ^fl/fl^ mice. (G) Pretreatment with WIN (i.pl.) 15 min alleviated GMQ‐induced mechanical allodynia in WT but not in *Trpv1*;*Cnr1* ^fl/fl^ mice. (B–G) Two‐way ANOVA with Sidak correction for multiple comparisons or One‐way ANOVA with Tukey's multiple comparisons test; ^*^ *p* < 0.05, ^**^ *p* < 0.01, ^***^ *p* < 0.001, ^****^ *p* < 0.0001. Sample sizes are indicated in parentheses. (H) Strategy of constructing *Trpv1*;iDTR mice. (I,J) Immunofluorescence verification of TRPV1^+^ neuron ablation in DRG. Scale bar = 50 µm, 20 × objective, zoom = 1.28 ×. Unpaired t tests; n = 6 mice/group; 4–5 sections/mouse. (K) GMQ‐induced licking behaviors attenuated in *Trpv1*;iDTR mice. Unpaired t tests; n = 10 mice/group; means ± SEM. M, male; F, female; BL, baseline; Veh, vehicle; WIN, WIN 55,212‐2.

In order to further investigate whether isolated ASIC3 activation produces pain behaviors similar to those observed in postoperative mice and whether CB_1_R activation can functionally antagonize ASIC3‐driven nociception in vivo, we employed the ASIC3‐selective agonist GMQ (Figure 3A), which opens the channel independently of pH changes [22], thereby isolating ASIC3‐specific behavioral responses. Intraplantar injection of GMQ evoked dose‐dependent licking behavior (Figure 3B) and mechanical allodynia (Figure 3C) in WT mice, responses that were equivalent in males and females (Figure S5A). Importantly, at the same dose, 60 nmol GMQ did not induce any significant licking behavior (Figure S5B) or mechanical allodynia (Figure S5C) in the ASIC3 KO mice, confirming that GMQ‐induced pain behavior in WT mice at this dose is ASIC3‐dependent. These data establish that ASIC3 activation is sufficient for key features of postoperative pain hypersensitivity. We then examined the effect of peripheral CB_1_R activation on GMQ‐induced nociception. Notably, intraplantar injection of WIN dose‐dependently suppressed GMQ‐induced licking behavior (Figure 3D), and this inhibition was completely reversed by the CB_1_R antagonist AM251 (Figure S5D), demonstrating CB_1_R dependence. WIN also significantly attenuated GMQ‐induced mechanical allodynia in WT mice (Figure S5E).

To determine whether nociceptor CB_1_R is required for this functional antagonism, we next examined conditional knockout lines. The inhibitory effect of WIN on GMQ‐evoked licking was entirely lost in both *Adv;Cnr1^fl/fl^* mice and *Trpv1;Cnr1^fl/fl^* mice (Figure 3E,F). Consistently, WIN prevented GMQ‐induced mechanical allodynia in WT mice, but not in *Trpv1;Cnr1^fl/fl^* mice (Figure 3G). These data indicate that CB_1_R on TRPV1^+^ nociceptors is required for the in vivo suppression of ASIC3‐mediated pain. To further confirm the contribution of TRPV1^+^ nociceptors we used *Trpv1;*iDTR mice, in which diphtheria toxin (DTX) administration selectively ablates TRPV1^+^ neurons (Figure 3H–J). In these mice, GMQ‐induced licking behavior was profoundly attenuated compared with controls (Figure 3K), directly demonstrating that ASIC3‐driven nociceptive responses require TRPV1‐expressing afferents. Collectively, these results demonstrate that CB_1_R activation in TRPV1^+^ nociceptors functionally inhibits ASIC3‐mediated pain behaviors, establishing a direct in vivo antagonism between peripheral cannabinoid signaling and ASIC3‐dependent nociception.

### 2.4 CB_1_R and ASIC3 are Co‐Expressed in CGRP^+^ Peptidergic Nociceptors

**FIGURE 4:**
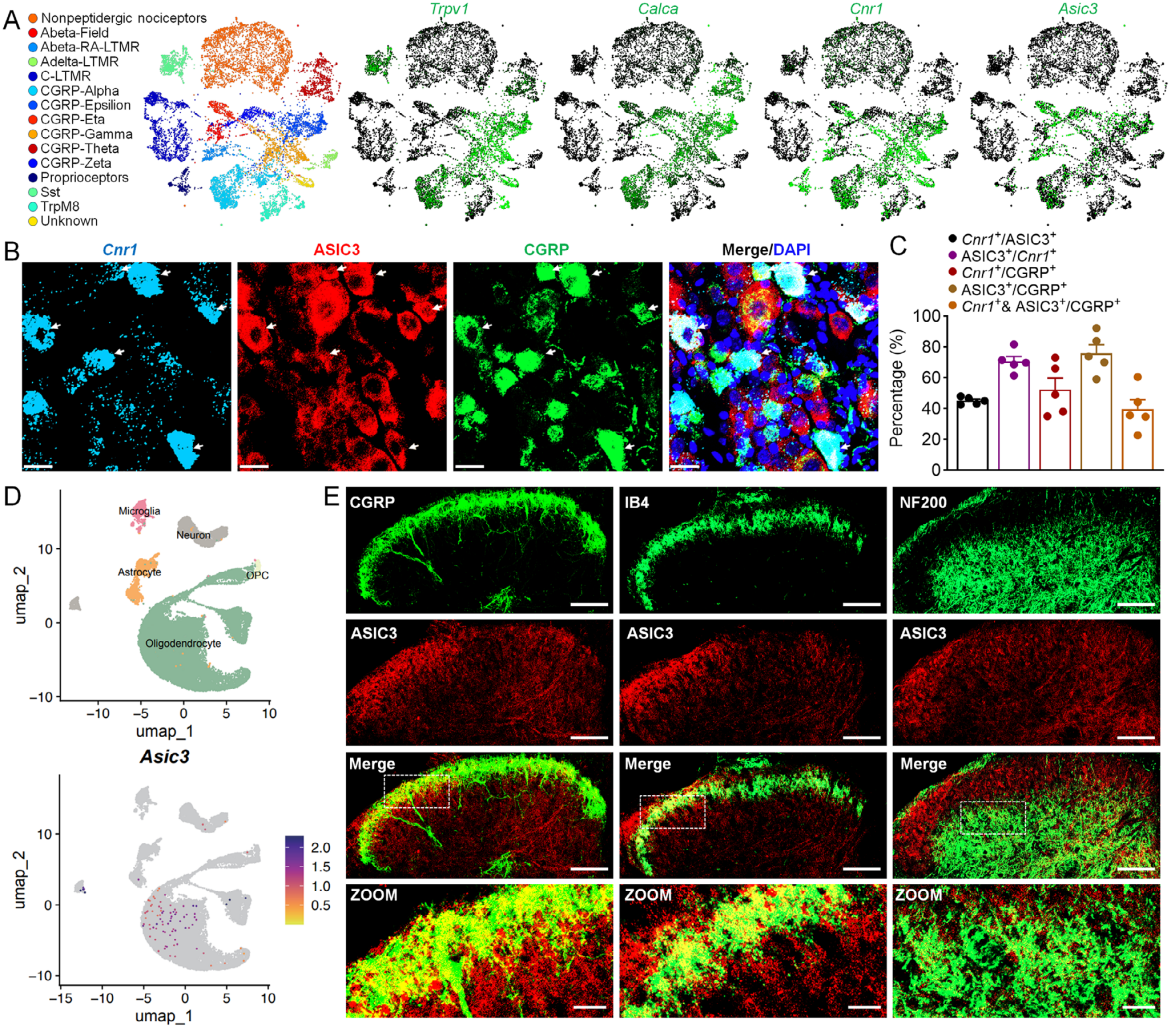
CB_1_R and ASIC3 are co‐expressed in CGRP^+^ neurons. (A) t‐SNE visualizations *Trpv1*, *Calca* (encoding CGRP), *Cnr1*, and *Asic3* expression in adult mouse DRG neurons, based on a published single‐cell RNA‐seq dataset (https://kleintools.hms.harvard.edu/tools/springViewer_1_6_dev.html?datasets/Sharma2019/adult). (B,C) Representative combined ASIC3/CGRP immunofluorescence and *Cnr1* RNAscope in situ hybridization images in DRG sections and quantification. Scale bars = 20 µm, 20 × objective, zoom = 2.84 ×; n = 5 mice/group, 3–5 sections/mouse. (D) UMAP plot revealing the *Asic3* gene is rarely expressed in cells of the mice spinal cord. (E) Immunofluorescence staining of the spinal dorsal horn from WT mice for ASIC3 together with IB4, CGRP, and NF200. Scale bars = 100 µm, 20 × objective. Zoomed‐in views of the boxed regions, scale bars = 20 µm, zoom = 4.25 ×.

The behavioral data above demonstrate that CB_1_R activation functionally inhibits ASIC3‐mediated nociception in vivo. To define the cellular basis for this functional interaction, we next examined whether CB_1_R and ASIC3 are co‐expressed in the same population of sensory neurons. We first analyzed a published single‐cell RNA‐sequencing (scRNA‐seq) dataset of adult mouse DRG neurons [23], t‐SNE visualization reveals that *Cnr1* and *Asic3* transcripts are predominantly co‐expressed in the CGRP^+^ (*Calca* ^+^) peptidergic subpopulation of *Trpv1* ^+^ nociceptors (Figure 4A). Consistent with this result, another single‐cell sequencing study of the nervous system indicates that the sensory neurons *Cnr1* and *Asic3* are also co‐expressed in the *Calca* ^+^ subpopulation (Figure S6A) [24]. This co‐localization within a defined nociceptor subset provides the cellular rationale for investigating the CB_1_R‐ASIC3 interaction.

We validated this co‐expression using combined immunofluorescence and RNAscope in situ hybridization in DRG sections. Quantitative analysis shows that 45% of ASIC3^+^ neurons express *Cnr1* mRNA, and 71% of *Cnr1* ^+^ neurons are ASIC3‐immunoreactive (Figure 4B,C). Within the CGRP^+^ peptidergic population, 52% of neurons express *Cnr1*, 76% express ASIC3, and a substantial subset (40%) co‐expresses both (Figure 4C). The size distribution profiles of ASIC3^+^, *Cnr1* ^+^, and CGRP^+^ neurons overlap considerably (Figure S6B), consistent with their predominant localization in small‐ to medium‐diameter nociceptors. We also analyzed the spinal distribution of ASIC3. UMAP analysis of mouse spinal cord scRNA‐seq data reveals that *Asic3* transcripts are virtually absent from spinal cord cells (Figure 4D), whereas *Asic1* and *Asic2* are readily detected (Figure S6C), confirming that ASIC3 is mainly expressed in the peripheral nervous system. Immunofluorescence staining of spinal dorsal horn sections demonstrates that ASIC3 protein is enriched in superficial laminae and colocalizes extensively with CGRP^+^ peptidergic terminals, and to a lesser extent with IB4^+^ non‐peptidergic terminals and NF200^+^ myelinated afferents, but is absent from spinal cord neurons (Figure 4E). Together, these results established that CB_1_R and ASIC3 are prominently co‐expressed in CGRP^+^ peptidergic nociceptors, providing a direct cellular basis for their functional interaction in the peripheral regulation of postoperative pain.

### 2.5 CB_1_R Physically Interacts With ASIC3 and Suppresses its Channel Activity and Downstream ERK_1/2_ Signaling

**FIGURE 5:**
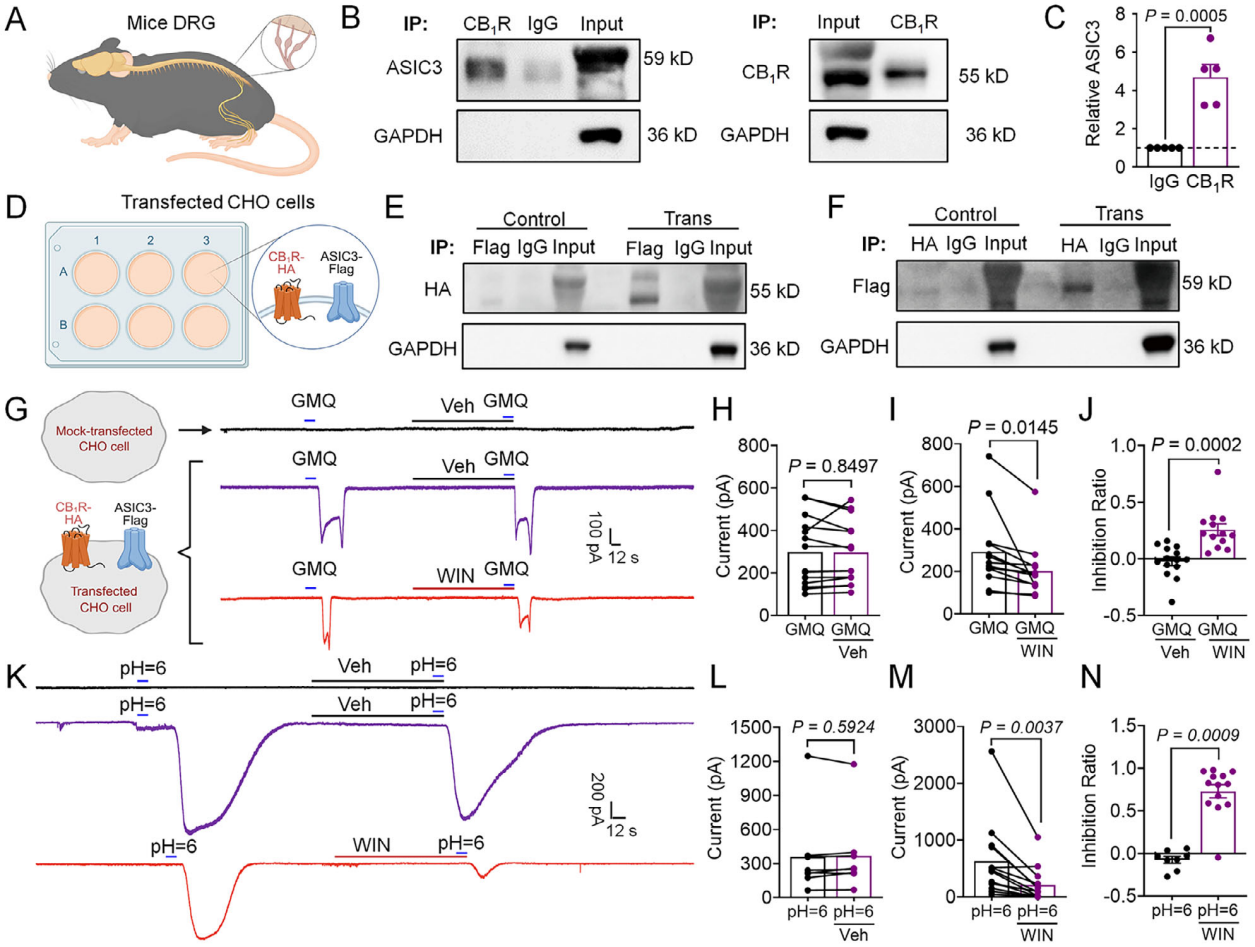
CB_1_R physically interacts with ASIC3 and inhibits its channel function. (A‐C) Co‐immunoprecipitation (Co‐IP) of endogenous CB_1_R and ASIC3 proteins from WT mouse DRG lysates. Unpaired t tests; n = 5 samples/group; 3 mice/sample. (D‐F) Reciprocal Co‐IP of HA‐tagged CB_1_R and Flag‐tagged ASIC3 heterologously expressed in CHO cells. (G‐J) Representative traces and quantifications of pretreatment with 10 µM WIN (2 min) to 3 mM GMQ (10 s)‐induced inward current in CHO cells. Paired/unpaired t tests; n = 13–14 cells/groups. (K‐N) Representative traces and quantifications of pretreatment with 10 µM WIN (2 min) to the extracellular solution at pH 6.0‐induced inward current in CHO cells. Paired/unpaired t tests; n = 8–13 cells/groups; means ± SEM. Veh, vehicle; WIN, WIN 55,212‐2.

Having established that CB_1_R and ASIC3 are co‐expressed in CGRP^+^ peptidergic nociceptors, we next investigated the molecular basis of their functional interaction. To determine whether CB_1_R physically associates with ASIC3, we performed co‐immunoprecipitation (Co‐IP). Endogenous CB_1_R and ASIC3 reciprocally co‐precipitated from mouse DRG lysates (Figure 5A–C), and heterologously expressed HA‐tagged CB_1_R and Flag‐tagged ASIC3 similarly co‐precipitated in CHO cells (Figure 5D–F), indicating that the two proteins associate within a protein complex in native sensory neurons and in a reconstituted system. To determine the functional consequences of this interaction, we used whole‐cell patch‐clamp recordings to measure inward currents in transfected CHO cells induced by the ASIC3‐specific agonist GMQ at pH 6.8. The results showed that in the cells co‐expressing ASIC3 and CB_1_R, perfusion with 3 mM GMQ induced an approximately 300 pA inward current that was absent in mock‐transfected controls (Figure 5G–I). Pretreatment with the ASIC3 selective blocker APETx2 (1 µM) significantly suppressed GMQ‐evoked inward currents (Figure S7A–C), confirming that GMQ‐induced currents are mediated specifically by ASIC3. Importantly, pretreatment with the CB_1_R agonist WIN (10 µM) also significantly suppressed the amplitude of GMQ‐induced currents (Figure 5G,J). To further simulate the opening of ASIC3 mediated by the decrease in tissue pH after an incision in vivo, as well as the regulatory effect of CB_1_R activation on ASIC3, we find that perfusion with an extracellular solution at pH 6.0 evokes robust inward currents in CHO cells co‐expressing ASIC3 and CB_1_R. Critically, preapplication of WIN significantly inhibits these proton‐evoked currents (Figure 5K–N), demonstrating that the CB_1_R functionally inhibits ASIC3 channel activity in response to GMQ and its native physiological agonist.

**FIGURE 6:**
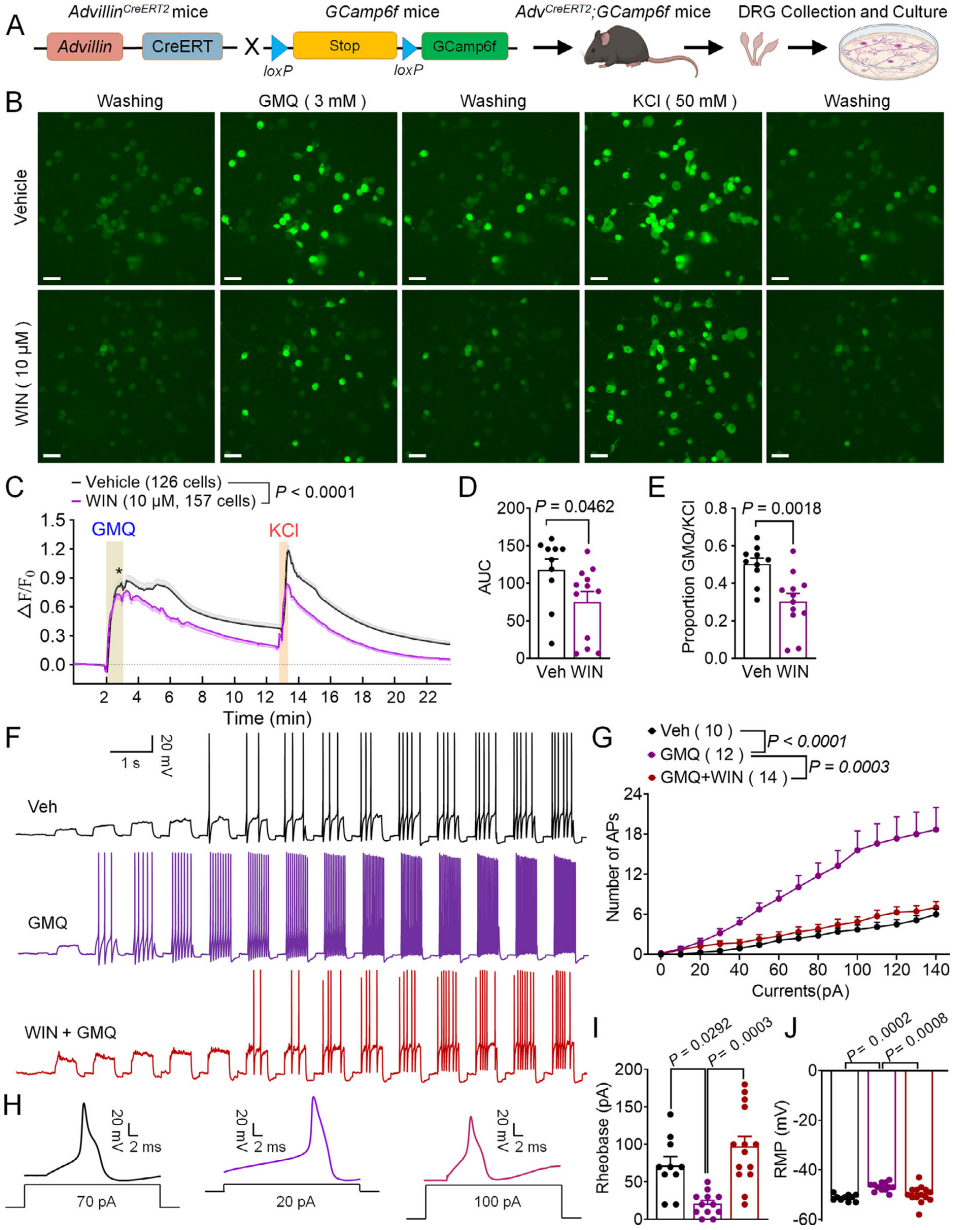
CB_1_R activation suppresses ASIC3‐mediated sensory neuron excitability. (A) Strategy of constructing *Adv;Rosa26^GCaMP6f^* mice for calcium imaging. (B) Representative traces of GMQ and KCl‐evoked calcium transients. Scale bars = 50 µm, 20 × objective, zoom = 1.3 ×. (C) ΔF/F0 traces from cultured DRG neurons of *Adv;Rosa26^GCaMP6f^* mice in response to sequential application of the ASIC3 agonist GMQ and KCl. (D) Quantification of the area under the curve (AUC) for GMQ‐evoked calcium transients. (E) Proportion of GMQ‐responsive neurons among total KCl‐responsive neurons. Unpaired t tests; n = 10–12 slides/group. (F,G) Representative action potential traces and quantification of firing frequency in cultured WT DRG neurons following treatment with GMQ (3 mM) alone or in combination with WIN (10 µM). Two‐way ANOVA with Sidak correction for multiple comparisons; n = 10–14 cells/group from 3 mice. (H,I) Representative traces and quantifications of rheobase of cultured WT DRG neurons. (J) Quantifications of resting membrane potentials (RMP) of cultured WT DRG neurons after different treatments. One‐way ANOVA with Tukey's multiple comparisons test; n = 10–14 cells/group from 3 mice; means ± SEM. Veh, vehicle; WIN, WIN 55,212‐2.

We next examined the functional consequences of this interaction on sensory neuron excitability. First, calcium imaging in cultured DRG neurons from *Advillin^CreERT2^;Rosa26^GCaMP6f^* mice showed that GMQ evoked robust calcium transients (Figure 6A–C). WIN pretreatment markedly reduced GMQ‐induced calcium signals and decreased the proportion of GMQ‐responsive neurons among KCl‐responsive neurons (Figure 6D,E), indicating that CB_1_R activation attenuates ASIC3‐mediated neuronal activation. Consistent with this, WIN preapplication significantly suppressed GMQ‐induced increases in action potential firing frequency (Figure 6F,G), elevated rheobase (Figure 6H,I), and hyperpolarized resting membrane potential (RMP, Figure 6J) in WT mice DRG neurons. Additionally, DRG neurons acutely isolated from incised vs. naïve mice were recorded to directly test whether incised DRG neurons exhibit detectably altered excitability. However, there are no significant differences between the two groups in action potential firing frequency, RMP, or rheobase (Figure S7D–H), which might be due to the fact that the DRG dissection itself constitutes a significant mechanical injury, which could potentially mask the more subtle in vivo changes induced by plantar incision. To validate the ASIC3‐dependence of the excitability changes in DRG neurons, ASIC3 blocker APETx2 was applied and the results demonstrated that APETx2 (1 µM) significantly suppressed GMQ‐induced increases in action potential firing frequency, elevated rheobase, and hyperpolarized RMP in wild‐type DRG neurons (Figure S7I–M). Conversely, DRG neurons from *Trpv1;Cnr1^fl/fl^* mice displayed intrinsic hyperexcitability, which was normalized by the ASIC3 blocker APETx2 (Figure S8A–E), confirming that ASIC3 is a key mediator of CB_1_R‐dependent control of neuronal excitability.

**FIGURE 7:**
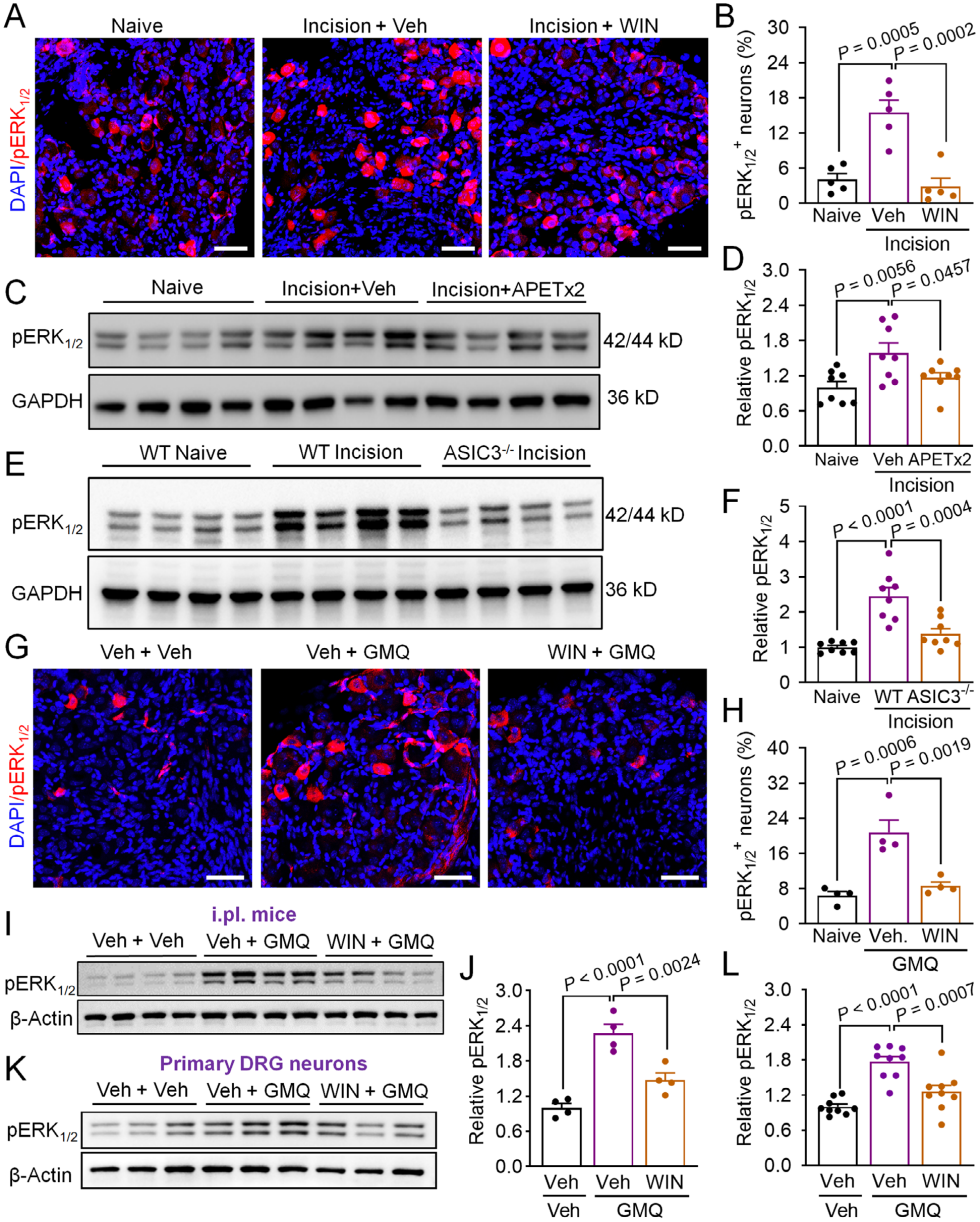
CB_1_R inhibits incision and ASIC3‐mediated ERK_1/2_ phosphorylation. (A,B) Representative pERK_1/2_ immunostaining images and quantifications in DRG sections of WT mice on postoperative day 1 and treated with intraplantar injection of WIN. Scale bar = 50 µm, 20 × objective, zoom = 1.28 ×. n = 5 mice/group, 3–4 sections/mouse. (C,D) Western blot analysis and quantification of pERK_1/2_ in DRG from mice treated with intraplantar injection of APETx2 on postoperative day 1. n = 8 mice/group. (E,F) pERK_1/2_ levels in DRGs from WT and ASIC3 KO mice on postoperative day 1. n = 8 mice/group. (G,H) Representative pERK_1/2_ immunostaining and quantification in DRG after intraplantar injection of GMQ 30 min. Scale bar = 50 µm, 20 × objective, zoom = 1.46 ×. n = 4 mice/group, 4–5 sections/mouse. (I,J) Western blot analysis of pERK_1/2_ in DRG from mice treated with GMQ alone or in combination with WIN. n = 4 mice/group. (K,L) pERK_1/2_ levels in cultured DRG neurons stimulated with 3 mM GMQ in the presence or absence of 10 µM WIN. n = 9 wells/group. One‐way ANOVA with Tukey's multiple comparisons test; means ± SEM. Veh, vehicle; WIN, WIN 55,212‐2.

We next asked whether this inhibition propagates to intracellular signaling cascades. Postoperative pain is associated with ERK_1/2_ phosphorylation in DRG neurons [25, 26], a marker of nociceptive activation. Intraplantar injection of WIN at postoperative day 1 markedly suppressed the incision‐induced increase in phosphorylated ERK_1/2_ (pERK_1/2_) in DRG neurons (Figure 7A,B), directly demonstrating that peripheral CB_1_R activation inhibits incision‐evoked pERK_1/2_ signaling. Moreover, incision‐induced pERK_1/2_ upregulation in the DRG was reversed by APETx2 in mice (Figure 7C,D). Consistently, the incision‐induced pERK_1/2_ upregulation was absent in ASIC3 KO mice and rats (Figure 7E,F and Figure S9A,B), confirming that incision‐induced ERK_1/2_ phosphorylation is ASIC3‐dependent. Furthermore, direct activation of ASIC3 by intraplantar GMQ was sufficient to elevate pERK_1/2_ in the DRG (Figure 7G,H) and intraplantar pre‐injection of the ERK inhibitor PD98059 (10 µg) significantly attenuated GMQ‐induced mechanical allodynia (Figure S9C), suggesting that ERK_1/2_ phosphorylation is functionally necessary for the ASIC3‐mediated pain behavior. Critically, WIN treatment significantly attenuated both GMQ‐induced pERK_1/2_ elevation in vivo (Figure 7G–J) and GMQ‐stimulated pERK_1/2_ in cultured DRG sensory neurons (Figure 7K,L). Collectively, these findings demonstrated that CB_1_R physically couples to ASIC3, inhibits its channel activity and downstream calcium‐ERK_1/2_ signaling, and thereby constrains the excitability of nociceptive sensory neurons.

### 2.6 Targeting the CB_1_R‐ASIC3 Axis Accelerates the Resolution of Postoperative Pain

**FIGURE 8:**
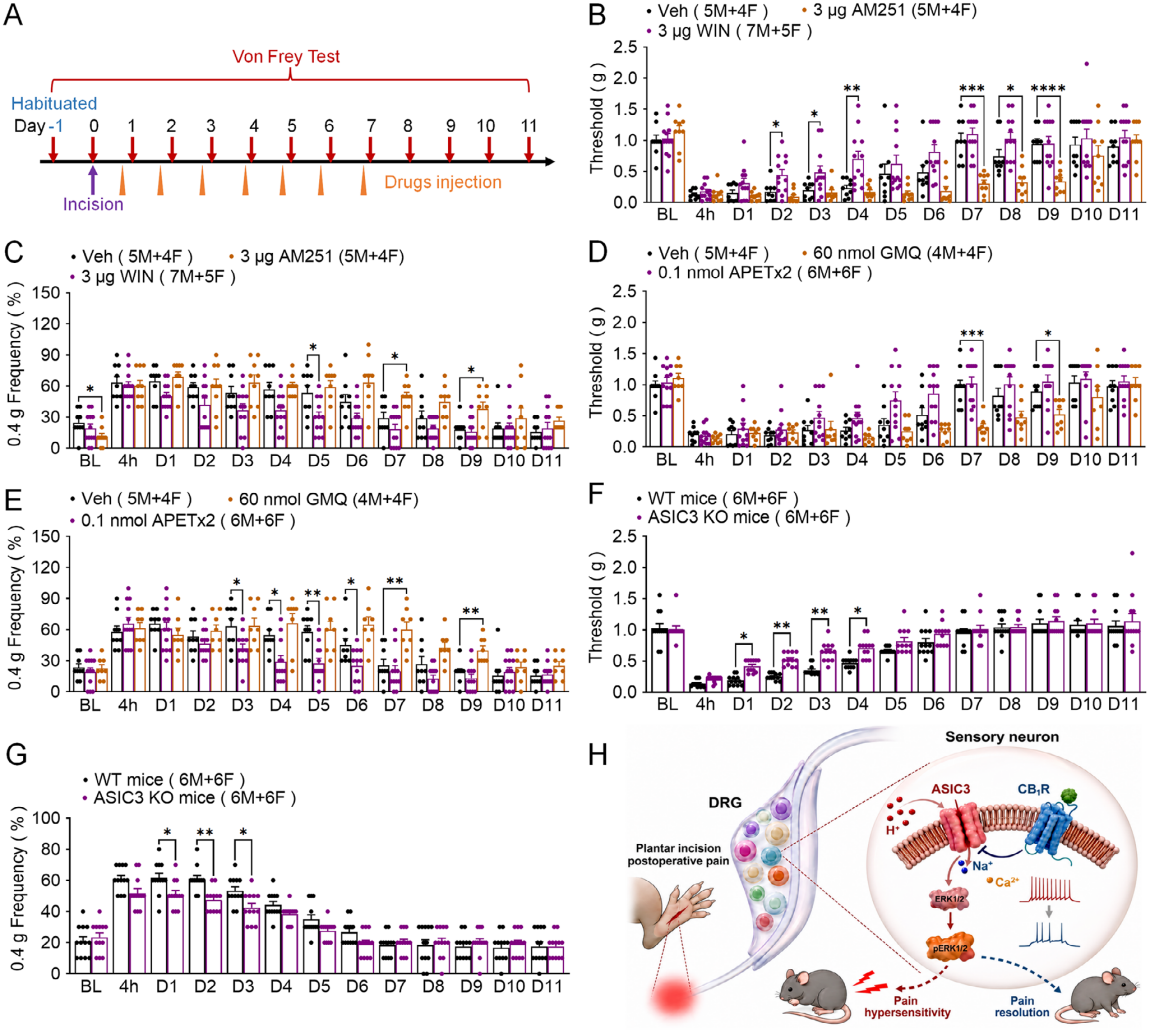
Repeated targeting of the CB_1_R‐ASIC3 axis accelerates the resolution of incision‐induced postoperative pain. (A) Experimental timeline depicting plantar incision, daily intraplantar drug administration, and behavioral assessment. (B,C) Mechanical paw withdrawal threshold (PWT) and response frequency to a 0.4 g von Frey filament in mice receiving daily intraplantar injection of the CB_1_R agonist WIN or the CB_1_R antagonist AM251. (D,E) PWT and 0.4 g‐evoked response frequency in mice treated daily with the ASIC3 blocker APETx2 or the ASIC3 agonist GMQ. (F,G) PWT and 0.4 g‐evoked response frequency in WT mice and ASIC3 KO mice following plantar incision. Two‐way ANOVA with Sidak multiple comparisons. ^*^ *p* < 0.05, ^**^ *p* < 0.01, ^***^ *p* < 0.001, and ^****^ *p* < 0.0001. Sample sizes are indicated in parentheses; means ± SEM. M, male; F, female; BL, baseline; Veh, vehicle; WIN, WIN 55,212‐2. (H) Proposed model of CB_1_R‐mediated postoperative analgesia in nociceptors through ASIC3 inhibition. In the mouse DRG, CB_1_R and ASIC3 are prominently co‐expressed in CGRP‐positive nociceptors. Plantar incision triggers an upregulation of ASIC3 and subsequent activation of ASIC3‐expressing neurons, thereby driving postoperative mechanical allodynia. Activation of nociceptor CB_1_R suppresses both ASIC3 channel function and ASIC3‐driven ERK_1/2_ phosphorylation, thereby braking nociceptive signaling and accelerating pain resolution. This intrinsic CB_1_R‐ASIC3 regulatory module thus represents a pivotal endogenous analgesic mechanism and a promising therapeutic target for the management of postoperative pain. H, Created with BioRender.com, https://BioRender.com/eft35rm.

Finally, we assessed whether sustained pharmacological engagement of CB_1_R‐ASIC3 axis could not only attenuate pain but actively promote its resolution, a clinically desirable endpoint that reflects restoration of normal sensory function rather than mere symptom suppression. To test this, we employed repeated daily intraplantar drug administration during the postoperative recovery phase (Figure 8A).

Repeated injection of the CB_1_R agonist WIN 55,212‐2 significantly accelerated the recovery of mechanical paw withdrawal thresholds and reduced response frequencies to a 0.4 g von Frey filament, whereas the CB_1_R antagonist AM251 aggravated mechanical allodynia and prolonged recovery (Figure 8B,C). Mirroring this pattern, daily blockade of ASIC3 with APETx2 promoted pain resolution, while daily ASIC3 activation with GMQ delayed recovery (Figure 8D,E). Congruently, ASIC3 KO mice (Figure 8F,G) and ASIC3 KO rats (Figure S9D) resolved incision‐induced mechanical allodynia faster than WT controls. These data establish that repeated targeting of the CB_1_R‐ASIC3 axis accelerates the resolution of acute postoperative mechanical hypersensitivity, and this finding raises the possibility that this pathway could be relevant for preventing prolonged pain states.

## 3 Discussion

Cannabinoids have emerged as attractive alternatives or supplements to therapy with opioids for chronic pain states. However, their clinical utility is limited by centrally mediated psychotropic side effects, a direct consequence of broad CB_1_R activation within the brain [6, 27, 28, 29]. Notably, postoperative pain, distinct from chronic pain in its etiology, is driven primarily by peripheral nociceptor activation rather than central sensitization. Primary sensory neurons of the DRG serve as the primary relay for transmitting noxious signals from surgical sites to the spinal cord. The present study identifies a peripheral CB_1_R‐ASIC3 signaling axis in peptidergic nociceptors that underlies cannabinoid‐mediated postoperative analgesia and governs the resolution of incisional pain, providing a compelling rationale for developing blood‐brain‐barrier‐impermeant CB_1_R agonists as a new class of postoperative analgesics.

In this study, we demonstrated that localized activation of CB_1_R on peripheral sensory neurons significantly alleviates mechanical allodynia in a murine postoperative pain model. Conversely, conditional deletion of CB_1_R in these neurons resulted in exaggerated basal mechanical sensitivity, while thermal nociception remained intact, suggesting that CB_1_R signaling selectively gates mechanosensitive pathways in the DRG. This phenotype aligns with previous reports of tactile allodynia in *Nav1.8^Cre^*;*Cnr1* ^fl/fl^ mice [30], and unaltered thermal responses in *Pirt^Cre^;Cnr1* ^fl/fl^ mice [31]. Beyond these genetic models, pharmacological studies have shown that systemic blockade of CB_1_R and CB_2_R prevents the spontaneous resolution of incisional pain and prolongs spinal neuroinflammatory responses [8], and that the analgesic action of acetaminophen in incisional pain involves CB_1_R activation, likely at a supraspinal or spinal site [9]. These studies collectively underscore the importance of CB_1_R signaling in postoperative pain but focus primarily on central and spinal mechanisms. Our findings focused on sensory neurons and demonstrated that CB_1_R on peripheral peptidergic nociceptors, acting through physical and functional coupling to ASIC3, constitutes an entirely peripheral, neuron‐intrinsic analgesic mechanism that operates independently of spinal glial modulation.

As the comprehensive reviews have articulated, ASICs are increasingly recognized as dual‐function proteins that respond to chemo‐stimulation (proton sensing) and might be involved in mechanical sensitivity, but through fundamentally distinct mechanisms [11]. ASIC3 is recognized as contributing to mechanosensation may through the tether model of mechanotransduction, in which force is transmitted via extracellular matrix and cytoskeletal linkages [11, 32]. The activity of ASIC3 has been reported to be modulated by shear force at low pH or in the presence of non‐proton ligands [33]. Additionally, ASIC3 has been specifically implicated in dynamic mechanosensitivity of proprioceptors [34, 35], and its mechanosensory function is modulated by an acidic microenvironment [36], a condition characteristic of surgical incision. Consistent with this modality‐specific role, ASIC3 in muscle afferents has been shown to mediate mechanical, but not heat, hyperalgesia in a model of muscle inflammation [37]. Therefore, the selective functional coupling of CB_1_R to ASIC3 provides a plausible mechanistic basis for the modality‐specific phenotype that CB_1_R activation suppresses mechanical, but not thermal pain, by inhibiting this ion channel involved in mechanical sensitivity. The observation that peripheral *Cnr1* deletion upregulates ASIC3 protein in the DRG further indicates that tonic endocannabinoid signaling constitutively suppresses ASIC3 expression. The resultant ASIC3 hyperactivity may account for the basal mechanical hypersensitivity in mice that conditionally deleted *Cnr1* in sensory neurons, and this is supported by our finding that APETx2 reverses this hypersensitivity.

The rapid transduction of noxious stimuli post‐injury is largely mediated by ion channels. ASIC3, a proton‐gated channel abundant in DRG neurons, is a key sensor of tissue acidosis and a contributor to pain behaviors [38, 39]. Previous study established the functional importance of ASIC3 in postoperative pain, demonstrating that ASIC3 is upregulated in rat DRG after plantar incision and that its pharmacological blockade or siRNA‐mediated knockdown reduces pain behaviors [13]. Here, we demonstrate that plantar incision activates ASIC3^+^ neurons and that local ASIC3 blockade or global knockout significantly alleviates postoperative pain. Using the nonproton activator GMQ, we confirmed that ASIC3 activation alone is sufficient to elicit spontaneous pain and mechanical allodynia. The use of GMQ was critical for isolating ASIC3‐specific behavioral and cellular responses independently of pH changes, thereby avoiding confounding effects from other acid‐sensitive channels. Crucially, we establish a functional inhibitory relationship, showing that CB_1_R activation suppresses ASIC3‐mediated pain behaviors and GMQ‐evoked currents.

Mechanistically, the physical CB_1_R‐ASIC3 association enables rapid, spatially restricted inhibition of channel activity, while the suppression of the ASIC3‐Ca^2+^‐ERK_1/2_ cascade couples channel inhibition to reduced neuronal excitability and attenuated nociceptive transmission. The CB_1_R‐ASIC3 association extends prior work showing that GPCRs modulate sensory neuron ion channels through both the canonical G_i/o_‐cAMP‐PKA cascade and protein‐complex formation. While earlier studies demonstrated that P2X3, TRPV1, and voltage‐gated sodium channels are regulated by GPCR intracellular pathways [40, 41], our findings provide a conceptual framework that a GPCR, CB_1_R, physically associates with an ASIC channel family member, ASIC3, and this association is functionally linked to channel inhibition. While CB_1_R is known to modulate HCN channels [17], P2X3 receptors [16], and voltage‐gated calcium channels [42, 43]. Our findings identify ASIC3 as a previously unrecognized effector of this broader analgesic network, which is particularly relevant given the acidic microenvironment of the surgical wound. However, the structural basis underlying the CB_1_R‐ASIC3 association remains to be defined in the future. This physical association represents a plausible mechanism for functional inhibition. Beyond this, canonical CB_1_R signaling pathways may also contribute, such as the inhibition of adenylate cyclase and a reduction in cAMP levels, which play important roles in several aspects of cannabinoid function, including modulation of the conductance of voltage‐dependent K^+^ and Ca^2+^ channels [44, 45]. Phosphorylation mechanisms may also be involved. Previous studies have indicated that PKA‐dependent phosphorylation regulates ASIC currents in cortical neurons [46]. Other studies have shown that PKA phosphorylates ASIC1 at Ser479 in the intracellular C‐terminal tail and that JNK probably directly phosphorylates ASIC1b and ASIC3 in sensory neurons [47, 48]. Thus, PKA and JNK may be involved in the inhibitory effect of CB_1_R activation on ASIC3 currents. To further explore the relative contributions of physical association and second‐messenger signaling, experiments such as pertussis toxin‐mediated G_i/o_ uncoupling, or the use of CB_1_R mutants defective in G‐protein coupling while retaining physical association, will be necessary in future research.

Anatomically, ASIC3 and CB_1_R are highly expressed in DRG neurons, as reported in prior studies [30, 49]. The combination of conditional knockout strategies targeting distinct neuronal populations allowed precise genetic dissection of the nociceptors. Importantly, the scRNA‐seq analysis and anatomical co‐localization indicated substantial co‐expression of CB_1_R and ASIC3 within CGRP^+^ peptidergic nociceptors, a population critical for pain signal transmission from the periphery to the central nervous system. Consistently, recent studies have demonstrated that ASIC3 activation triggers CGRP release from sensory neurons, promoting neurogenic inflammation [50, 51, 52], underscoring the functional importance of ASIC3 in these neurons. Additionally, the conditional *Cnr1* deletion in sensory neurons substantially narrows the relevant cell population, yet it does not entirely exclude contributions from non‐neuronal CB_1_R. A modulatory role for CB_1_R on other local cell types warrants future investigation.

Although this study demonstrated the pivotal role of the CB_1_R‐ASIC3 axis in postoperative pain, the following limitations remain: (1) the precise structural basis of the CB_1_R‐ASIC3 interaction remains to be defined. Co‐IP does not distinguish direct binding from association within a larger complex. Proximity‐based assays such as PLA, FRET, or BRET can confirm close spatial proximity. Future work employing truncation mutagenesis and high‐resolution structural approaches will be required to map the interacting domains; (2) our experiments cannot fully dissociate the effects of physical coupling from canonical G‐protein‐dependent signaling; the relative contributions of direct interaction vs. downstream pathways such as cAMP/PKA and JNK remain to be evaluated, ideally through the use of interaction‐deficient CB_1_R mutants; (3) our pharmacological studies rely on WIN 55,212‐2, confirmation with peripherally restricted CB_1_R agonists is necessary to validate translational feasibility; (4) the study focuses exclusively on acute incisional pain in rodents; whether the CB_1_R‐ASIC3 axis remains operative in chronic postsurgical pain models or in human DRG tissue requires investigation.

In summary, our findings demonstrated that peripheral CB_1_R expressed in nociceptive DRG neurons plays essential roles in regulating physiological mechanical sensitivity and pathological postoperative pain. We have identified a novel peripheral analgesic pathway wherein CB_1_R on peptidergic nociceptors functionally and physically couples to ASIC3, inhibiting its channel activity, downstream calcium signaling, and ERK_1/2_ phosphorylation (Figure 8H). These findings nominate the peripheral CB_1_R‐ASIC3 signaling axis as a candidate target and provide a mechanistic foundation for developing peripherally acting non‐opioid analgesic strategies with favorable safety profiles for postoperative pain management.

## 4 Materials and Methods

### 4.1 Animals

In the present study, 10–12 weeks old male and female mice of the C57BL/6 genetic background were utilized. *Advillin^CreERT2^* mice (stock no. 026516), *Trpv1^Cre^* (stock no. 017769), *Cnr1* ^flox/flox^ mice (stock no. 036107), CB_2_R^−/−^ mice (stock no. 005786), R26^iDTR^ mice (stock no. 007900) and C57BL/6J wild‐type mice (stock no. 000664) were purchased from the Jackson Laboratory (USA). Rosa26^GCaMP6f^ mice were purchased from Cyagen Biosciences (Guangzhou, China). In order to investigate the role of ASIC3 in postoperative pain, ASIC3 global knockout (ASIC3 KO) mice and rats were also utilized. ASIC3 KO mice were gifted by Professor Tian‐Le Xu (Department of Anesthesiology, Songjiang Hospital and Songjiang Research Institute, Shanghai Jiao Tong University School of Medicine). ASIC3 KO rats on a Sprague‐Dawley background were generated via a CRISPR‐Cas9 system by Bioray Laboratories (China) as previously described [49]. All animals were housed under SPF conditions at the Southern University of Science and Technology Animal Facility and maintained on a 12‐h light/12‐h dark cycle at 22°C ± 1°C with ad libitum access to food and water. All experimental procedures were approved by the Laboratory Animal Welfare and Ethics Committee of the Southern University of Science and Technology (SUSTech‐JY202409029, Shenzhen, China) and were conducted in accordance with the ARRIVE guidelines and EU Directive 2010/63/EU. The experimenters were blinded to the treatment groups and outcome assessments.

### 4.2 Conditional Knockout of CB_1_R

Conditional knockout of CB_1_Rs in primary sensory neurons was achieved via the Cre‐loxP system. *Advillin^CreERT2^;Cnr1^flox/flox^* mice and *Trpv1^Cre^;Cnr1^flox/flox^* mice were generated to delete CB_1_R specifically in most of the primary sensory neurons or TRPV1‐positive neurons, respectively. The Advillin^CreERT2^ line expresses a tamoxifen‐inducible iCre recombinase directed by mouse Advillin promoter elements [53], which is expressed almost exclusively in peripheral sensory neurons [54]. To induce recombination, tamoxifen (T5648, Sigma) was dissolved in corn oil and injected intraperitoneally (i.p.) at a dose of 150 mg/kg (20 mg/mL) every other day for a total of four injections. Genomic DNA was extracted from the tails or ears of the genetic mice via the alkaline lysis method. The tissue samples were incubated in 2.5 mM NaOH and 0.2 mM EDTA at 100°C for 1 h, followed by pH neutralization with 40 mM Tris‐HCl (pH 5.5) and centrifugation to collect the supernatant. Genotyping was performed via PCR primers. The PCR products were resolved on 1.5% agarose gels stained with nucleic acid dye. Behavioral experiments commenced 14 days after the final tamoxifen injection.

### 4.3 Ablation of TRPV1 Neurons

*Trpv1^Cre^*;iDTR mice were generated by crossing *Trpv1^Cre^* mice with R26^iDTR^ mice. To ablate TRPV1^+^ neurons, *Trpv1^Cre^*;iDTR mice were intraperitoneally (i.p.) injected with diphtheria toxin (DTX, D0564, Sigma, 50 µg/kg, 5 µg/mL in sterile PBS) every other day 2 times. Control *Trpv1^Cre^*;iDTR mice received PBS. The ablation efficiency was verified 2–4 days after the final injection, and subsequent experiments were performed 14 days post‐injection.

### 4.4 Plantar Incision‐Induced Postoperative Pain Model

Postoperative pain was induced via a previously established plantar incision model [55]. Briefly, the mice were anesthetized with 2% isoflurane, and the right hind paw was sterilized with 10% povidone‐iodine solution. A 5‐mm longitudinal incision was made through the skin, fascia, and underlying plantaris muscle from the proximal edge of the heel toward the toes (with the muscle origin remaining intact). Hemostasis was achieved by gentle pressure, and the wound was closed with two mattress sutures using 4‐0 nylon; antibiotic ointment was applied topically. Mechanical allodynia was assessed via von Frey filaments at 4 and 24 h postoperation, as previously described [56]. The analgesic effects of the drugs were measured at different time points after intraplantar (i.pl.) administration on postoperative day 1. The drugs used included the cannabinoid receptor agonist WIN 55212‐2 (GC37935, GlpBio), the CB_1_R selective antagonist AM251 (S2819, Selleck), the ASIC3 specific blocker APETx2 (ab141849, Abcam), the CB_2_R selective agonist AM1241 (S1544, Selleck), and MEK/ERK inhibitor PD98059 (HY‐12028, MedChemExpress). The above drugs were dissolved in a vehicle consisting of 5% DMSO, 5% castor oil, and 90% sterile saline. For chronic drug treatments, compounds are generally administered after behavioral testing.

### 4.5 GMQ (2‐Guanidine‐4‐Methylquinazoline) Test

The mice were individually habituated to small plastic chambers with metal mesh floors for 2 days. All behavioral experiments were conducted as previously reported [57]. In this experimental series, nociceptive behavior was induced by i.pl. a total of 10 µL of GMQ (SML0874, Sigma) was injected into the right hind paw via a 30 G needle. Licking behavior was video recorded for 30 min immediately after injection, and the time spent licking the injected paw was quantified via a stopwatch. To assess CB_1_R modulation of ASIC3‐mediated nociception, 3 µg of WIN 55212‐2 (10 µL) was injected i.pl. 15 min before GMQ injection. GMQ‐induced mechanical allodynia was also immediately measured at different time points after injection via von Frey filaments.

### 4.6 Tail‐Flick Test and Hot‐Plate Test

For tail‐flick test, mice were gently held by hand with a terry glove. The exposed distal part of the tail (2 cm) was immersed into 50°C hot water. The tail‐flick latency was defined as the time required for a mouse to flick or remove its tail out of hot water. A maximum cutoff value of 15 s was set to avoid thermal injury. For hot‐plate test, mice were placed on the hot‐plate at 53°C, and the reaction time was scored when the animal began to exhibit signs of pain avoidance such as paw licking/shanking or jumping. A maximum cutoff value of 40 s was set to avoid thermal injury.

### 4.7 Rotarod Test

To assess potential effects of intraplantar WIN administration on motor coordination and sedation, the rotarod test was performed as previously described [55]. Mice were trained to walk on a rotarod apparatus (16 rpm, ZB‐200, Chengdu Technology & Market Co., Ltd., Chengdu, China) for two consecutive days, with three training sessions per day and a cut‐off time of 300 s per trial. The rotarod drum was cleaned with 75% ethanol after each trial to eliminate olfactory cues. Mice that failed to remain on the rotating rod for at least 180 s during the final training session were excluded from further testing. On the third day, mice received an intraplantar injection of either vehicle or WIN 55 212‐2 (3 µg), and the latency to fall off the rod was recorded at 30, 60, 90, 120, 150, and 180 min post‐injection.

### 4.8 Open Field Test

To evaluate whether intraplantar WIN administration affects spontaneous locomotor activity, the open field test was performed. Mice were habituated to the testing environment for two consecutive days before the experiment. On the third day, mice received an intraplantar injection of vehicle or WIN (3 µg), and testing began 30 min after injection. Each mouse was individually placed in the center of an uncovered square arena (40 × 40 × 40 cm) with white opaque plexiglass walls. Spontaneous activity was recorded from a top‐down perspective for 60 min using an overhead video camera. After each trial, the arena was thoroughly cleaned with 75% ethanol to eliminate olfactory cues. Motion trajectories and time spent in the entire arena were analyzed using the ANY‐Maze software (Stoelting Co., United States).

### 4.9 Gait Analysis Test

To evaluate the effect of peripheral CB_1_R activation on functional recovery after plantar incision, gait analysis was performed using the VisuGait Animal Gait Analysis System (XR‐FP101, Shanghai Xinrui, China). Mice were habituated to the walking stage and testing environment for two consecutive days before the experiment. On the third day, baseline gait parameters were recorded prior to surgery. Plantar incision was then performed as described above. At 24 h post‐incision, gait analysis was conducted to assess incision‐induced functional deficits. Mice subsequently received an intraplantar injection of vehicle or WIN (3 µg), and gait parameters were re‐evaluated 1 h after injection. For each trial, the mouse was placed at one end of the walking stage and allowed to walk freely to the other end. The walking process was recorded by a high‐speed camera, and footprint information was automatically extracted by the system. Gait parameters, including stance time (the duration of time within a single gait cycle during which a paw is in contact with glass plate), max contact area (is defined as the maximum area of a paw that comes into contact with the glass plate), print area (measures the surface area of the complete paw print), and print length (describes the length (horizontal direction) of the complete paw print) were identified and analyzed using VisuGait software (Version 2.0).

### 4.10 Immunofluorescence Staining

Following the completion of behavioral testing on postoperative day 1, mouse tissues were harvested as specified below. Anesthetized mice were perfused with 20 mL of PBS followed by 40 mL of precooled 4% (w/v) paraformaldehyde fix solution (PFA). Lumbar DRGs (L4‐L6) and the spinal cord were collected, fixed in 4% PFA overnight and then transferred to 20% sucrose followed by 30% sucrose for 24 h at 4°C. The tissues were then embedded in optimal cutting temperature (OCT) solution. The DRGs and spinal cord segments were cut onto slides via a freezing microtome (RWD Minux FS800, China) at 15 and 30 µm thicknesses, respectively. The sections were blocked for 1 h in 5% donkey serum (SL050, Solarbio) with 0.1% Triton X‐100 and incubated overnight at 4°C with primary antibodies against ASIC3 (ASC‐018, Alomone), c‐Fos (226017, SYSY), TRPV1 (ab6166, Abcam), CGRP (ab47027, Abcam), IB4 (L2895, Sigma), NF200 (N0142, Sigma), and pERK_1/2_ (9101S, Cell Signaling Technology) at 4°C overnight. The sections were incubated overnight at 4°C with the following secondary antibodies: Cy3 donkey anti‐rabbit (711‐165‐152, Jackson), Cy3 donkey anti‐rat (712‐165‐153, Jackson), FITC‐conjugated donkey anti‐rabbit (711‐095‐152, Jackson), and FITC donkey anti‐mouse (715‐095‐150, Jackson). After three rinses, the sections were mounted with Fluoroshield containing DAPI (Sigma, F6057) and imaged via a Zeiss LSM980 confocal microscope system. Image analysis was performed via Zeiss ZEN 3.4 (Blue Edition) software.

### 4.11 RNAscope in Situ Hybridization

RNAscope in situ hybridization was performed as previously described [58]. The mice were perfused with PBS and 4% PFA. L4‐L6 DRGs were harvested and postfixed overnight at 4°C. In situ hybridization was performed according to the manufacturer's instructions for the RNAscope Multiplex Fluorescent Detection Reagents Kit v2 (323110, Advanced Cell Diagnostics). Mouse DRG sections of 15 µm were used for in situ hybridization to detect the expression of *Cnr1* (RNAscope Probe‐Mm‐*Cnr1* 420721) and *Trpv1* (RNAscope Probe‐Mm‐*Trpv1* 313331). For combined immunofluorescence and in situ hybridization, the sections were blocked with 5% donkey serum in 0.1% Triton X‐100 for 1 h at room temperature and incubated overnight at 4°C with antibodies against ASIC3 and CGRP, followed by secondary antibody incubation overnight at 4°C.

### 4.12 Primary DRG Neuron Culture

L3‐L6 DRGs were aseptically collected from WT mice, *Trpv1;Cnr1* ^fl/fl^ mice, and *Advillin^CreERT2^;Rosa26^GCaMP6f^* mice (5‐6 weeks) in HBSS (14175‐095, Gibco) and digested with collagenase A (0.2 mg/mL, Roche)/dispase‐II (3 mg/mL, Roche) at 37°C for 90 min. Dissociated neurons were washed twice with HBSS and resuspended in neurobasal medium (A3582901, Thermo Fisher) containing 10% FBS (10099133C, Thermo Fisher), 1% penicillin/streptomycin (15140122, Gibco), and 2% B27 (17504044, Thermo Fisher). The cells were plated on glass coverslips coated with 0.1 mg/mL poly‐D‐lysine (1 mg/mL, Sigma, St. Louis, MO) and were cultured overnight for calcium imaging assay or whole‐cell patch‐clamp recordings. To determine whether plantar incision alters the intrinsic excitability of acutely isolated DRG neurons, a shortened culture protocol was employed. At 24 h after plantar incision or under naïve conditions, DRGs were harvested from WT mice and dissociated. Following dissociation, neurons were plated on poly‐D‐lysine‐coated coverslips and allowed to adhere for 2 h at 37 °C in neurobasal medium. Whole‐cell patch‐clamp recordings were then performed immediately to minimize the loss of in vivo electrophysiological properties.

### 4.13 Transfection of Chinese Hamster Ovary (CHO) Cells

CHO cells (1×10^5^ cells) were cultured in DMEM supplemented with 10% fetal bovine serum and 1% penicillin/streptomycin at 37°C in 5% CO_2_. At 60% confluence, the cells were transfected with mouse *Cnr1* (pCMV‐HA‐Cnr1(mouse)‐Neo, NM_001355020.2, Miaoling Plasmid Platform, P58454) and mouse *Asic3* (pECMV‐Asic3‐m‐FLAG, Miaoling Plasmid Platform, P6435) via JetPRIME transfection reagent (Polyplus SA, France). The cells were harvested 48 h posttransfection for coimmunoprecipitation or patch‐clamp experiments.

### 4.14 Calcium Imaging Assay

Intracellular calcium mobilization was measured in cultured primary DRG neurons from *Advillin^CreERT2^;Rosa26^GCaMP6f^* mice treated with intraperitoneal tamoxifen. DRG neurons cultured on glass coverslips were incubated with vehicle or 10 µM WIN 55212‐2 for 30 min at 37°C. Coverslips were placed in a recording chamber and perfused with standard extracellular solution (10 mM HEPES, 5 mM KCl, 135 mM NaCl, 2 mM MgCl_2_, 2 mM CaCl_2_, and 10 mM glucose, pH 7.4) at a flow rate of 2–4 mL/min via a fluorescence imaging plate reader. Fluorescence was recorded via a Hamamatsu ORCA C13440‐20CU CMOS camera. ASIC3‐positive neurons were identified in response to 3 mM GMQ; 50 mM KCl served as a positive control. The data are expressed as the relative change in fluorescence (△F/F_0_), where F_0_ is the basal fluorescence and △F is the change in real‐time fluorescence compared with the basal fluorescence.

### 4.15 Whole‐Cell Patch‐Clamp Recordings

Whole‐cell patch‐clamp recordings were performed on CHO cells at room temperature as previously described [59]. The patch‐pipettes with a resistance of 6–8 MΩ were then filled with internal solution. The pipette mixture contained (in mM) 126 K‐gluconate, 10 NaCl, 1 MgCl_2_, 10 EGTA, 2 Na‐ATP and 10 HEPES and was adjusted to pH 7.4 with KOH. The external solution contained (in mM) 135 NaCl, 5 KCl, 2 CaCl_2,_ 2 MgCl_2_, 10 HEPES, and 10 glucose and was adjusted to pH 7.4 with NaOH. The osmolarity was adjusted to 330 mOsm/L with sucrose. Cells on glass coverslips were transferred to a recording chamber and continuously perfused with external solution. The cells were visualized via a 40X water‐immersion objective (SliceScope Pro 6000, Scientifica). pH 6.0 or GMQ at pH 6.8‐induced ASIC3 currents were recorded at a holding potential of −70 mV in voltage‐clamp mode. GMQ (10 s) and WIN or APETx2 (2 min) were bath applied via gravity perfusion. The inhibition ratio of GMQ‐evoked currents by WIN or APETx2 was calculated as (I_1_ − I_2_) / I_1_, where I_1_ is the peak current amplitude evoked by the initial GMQ application and I_2_ is the peak current amplitude evoked by GMQ following WIN or APETx2 pretreatment. Action potentials of primary DRG neurons were evoked by a set of stepped increasing currents (0 to 140 pA, 500 ms; in increments of 10 pA). Small‐diameter (≤25 µm) neurons were selected, and recordings were initiated when their resting membrane potential (RMP) was more negative than ‐40 mV. GMQ was applied by bath perfusion for 2 min before action potential recordings were initiated. RMP was recorded in the current patch model. Rheobase was evoked by a set of stepped increasing currents (0 to 200 pA, 30 ms; in increments of 10 pA) until it produced an action potential.

### 4.16 Coimmunoprecipitation (Co‐IP)

Mouse DRGs or transfected CHO cells were harvested, washed once in ice‐cold PBS and then transferred to NP‐40 lysis buffer (HY‐K1002, MCE) supplemented with protease and phosphatase inhibitors, ensuring the preservation of protein integrity. The DRGs were then homogenized via an automatic homogenizer and incubated on ice for 30 min. The lysates were subsequently centrifuged at 12 000 rpm for 15 min, and the supernatants were quantified via the BCA assay (Beyotime Biotechnology, P0010S). For Co‐IP, the lysates were incubated overnight at 4°C with an anti‐CB_1_R antibody (sc‐293419, Santa Cruz) or a mouse IgG control (sc2025, Santa Cruz) to promote optimal binding. The CHO cell lysates were incubated with anti‐HA‐Tag antibody (30702ES20, YEASEN), anti‐Flag‐Tag antibody (F1804, Sigma), or the corresponding IgG control (A1923, A7058, Beyotime). Protein A/G magnetic beads (HY‐K0202, MedChemExpress) were added and incubated overnight at 4°C to capture the antigen‒antibody complexes. The IP complexes were washed and eluted by adding Laemmli buffer (2X), followed by boiling for 5 min. Western blotting analysis was used to further detect the IP complexes.

### 4.17 Western Blotting Analysis

Primary DRG neurons from naïve WT mice were seeded in a 24‐well plate. After 24 h of seeding, the cells were treated with 3 mM GMQ alone or after 30 min of pretreatment with WIN 55212‐1 (10 µM). Mouse DRG or spinal cord tissues were harvested following the completion of behavioral testing on postoperative day 1. Total protein was extracted via RIPA lysis buffer (Beyotime Biotechnology, P0013B) containing protease inhibitors (Roche, complete protease inhibitor cocktail, 11697498001) and phosphatase inhibitors (Roche, PhosSTOP EASYpack inhibitor tablets, 4906845001), and the lysates were centrifuged at 12 000 rpm for 15 min at 4°C. Protein samples or Co‐IP eluates were separated via SDS‐PAGE via 4%–20% SurePAGE gels (GenScript, M00657) and transferred to PVDF membranes. The membranes were blocked with 5% skim milk for 2 h and incubated overnight at 4°C with primary antibodies against pERK_1/2_ (9101S, Cell Signaling Technology), p‐p38 (4511, Cell Signaling Technology), CB_1_R (sc‐293419, Santa Cruz), and ASIC3 (ASC‐018, Alomone). The PVDF membranes were subsequently incubated with secondary antibodies for 1 h at room temperature. The secondary antibodies used in this study included goat anti‐rabbit IgG H&L (HRP, ab205718, Abcam), goat anti‐mouse IgG H&L (HRP, ab205719, Abcam), and goat anti‐rabbit light chain HRP‐conjugated antibodies (A120‐113P, Bethyl). Relative protein expression levels were normalized to those of GAPDH (HRP‐60004, Proteintech) or β‐actin (4967, Cell Signaling Technology). Immunoreactive bands were detected by enhanced chemiluminescence reagents (New Cell Molecular Biotechnology Co., Ltd., P10200) and scanned on a chemiluminescence imaging system (Sagecreation, MiniChemi610 Plus). Band intensities were quantified via ImageJ software (NIH, Bethesda, MD, USA).

### 4.18 DRG Single‐Cell RNA‐Seq Data Reanalysis

Single‐cell RNA‐sequencing (scRNA‐seq) data from mouse DRG and spinal cord were obtained from the NCBI Sequence Read Archive (SRA) under BioProject accession number PRJNA438862 [60]. The SRA experiment accessions used in this study were SRX3809321 and SRX3809270 (DRG), and SRX3809269, SRX3809316, SRX3809317, SRX3809318, SRX3809319, SRX3809344, and SRX3809345 (spinal cord). Data analysis was performed in R with Seurat (v4.3.0.1) [61]. Quality control was performed to exclude low‐quality cells based on commonly used criteria: number of unique molecular identifiers (UMIs), number of detected genes, mitochondrial read percentage, and molecular complexity (log10 genes per UMI). After filtering, 2948 cells with 27 998 genes were retained for DRG, and 36 444 cells with 15 332 genes were retained for the spinal cord. To integrate datasets, we performed canonical correlation analysis (CCA)‐based anchor integration using the FindIntegrationAnchors and IntegrateData functions [61]. The integrated data were scaled and subjected to principal component analysis (PCA). Graph‐based clustering (FindNeighbors, FindClusters) and UMAP visualization were performed using the first 30 principal components. Cell clusters were annotated based on canonical marker genes. After annotation, 2189 DRG neurons and 30 769 cells of key spinal cord cell types were extracted for subsequent expression analysis of target genes (*Asic1*, *Asic2*, *Asic3*, *Cnr1*, *Calca*).

### 4.19 Statistical Analyses

All the experiments were randomized, and the animals were assigned randomly from multiple cages. Statistical analyses were performed via Prism GraphPad version 9.0 (GraphPad Software, San Diego, CA, USA). Data are presented as means with standard errors (means ± SEM). For Western blotting and immunohistochemical quantification, the intensity of the protein bands or fluorescence were quantified using ImageJ software (NIH, Bethesda, MD, USA), and data were normalized to the respective controls or to the mean of the control group within each independent experiment to account for inter‐experiment variability. For all animal experiments, at least four different mice per condition were used. For analysis of in vitro cell cultures, at least five independent biological experiments were performed. The n values in the electrophysiological experiments represent the number of neurons from at least three mice. Behavioral data, such as mechanical paw withdrawal thresholds and licking durations, were analyzed in their original units without transformation. Differences in two set comparisons were analyzed using unpaired or paired t test. For more than two groups, a one‐way ANOVA followed by Tukey test was used to assess the statistical significance. For multiple mixed comparisons, a two‐way ANOVA followed by Sidak's test was used. The criteria for statistical differences were: ns (not significant), *p* > 0.05, ^*^ *p* < 0.05, ^**^ *p* < 0.01, ^***^ *p* < 0.001, ^****^ *p* < 0.0001; and all of the sample sizes and the statistical details for each quantification can be found in the figure legends.

## Author Contributions

K. X. and Z. W. drafted the original and revised manuscript. Conceptualization was performed by K. X., Z. W., and C. W. Methodology was developed by K. X., Z. W., L. J., and H. Y. Data curation were carried out by K. X., L. J., H. Y., and X. C. Investigation were performed by K. X., L. J., H. Y., X. C., Q. H., H. H., J. W., L. S., S. F., Y. L., and X. T. Validation were performed by K. X., L. J., H. Y., and X. C. Formal analysis were conducted by K. X., L. J., H. Y., and X. C. Supervision and funding acquisition were carried out by Z. W., C. W., and H. W. Visualization was performed by K. X., L. J., and H. Y. Project administration were provided by Z. W. and C. W. All authors participated in reviewing and editing the manuscript, and approved the final version for publication.

## Funding

Shenzhen Medical Research Funding (C2301006, B2402043); National Natural Science Foundation of China (82571440); Guangdong Basic and Applied Basic Research Foundation (2023B0303010002, 2024A1515012625); China Postdoctoral Science Foundation (2025M782722).

## Ethics Statement

All animal experiments were approved by the Laboratory Animal Welfare and Ethics Committee of Southern University of Science and Technology (Shenzhen, China) under approval number SUSTech‐JY202409029. All procedures involving mice and rats were conducted in strict accordance with the ARRIVE guidelines and complied with the ethical standards of EU Directive 2010/63/EU for the protection of animals used for scientific purposes.

## Conflicts of Interest

The authors declare no conflicts of interest.

## Supporting information




**Supporting File**: advs77925‐sup‐0001‐SuppMat.docx.

## Data Availability

The data that support the findings of this study are available from the corresponding author upon reasonable request.
