# Supplementary material for: Cannabinoid CB1 Receptor in Nociceptors Mediates Postoperative Analgesia via ASIC3 Inhibition
Source: Adv Sci (Weinh). 2026 Sep 27:e77925. Online ahead of print. doi: 10.1002/advs.77925 (PMC13616247; doi:10.1002/advs.77925)
Supplement: Supplementary file 1 [file ADVS-9999-e77925-s001.docx]

**Cannabinoid CB1 Receptor in Nociceptors Mediates Postoperative Analgesia Via ASIC3 Inhibition**

Kangtai Xu^1, 2, 3^, Luyao Ji^3^, Haoyi Yang^1, 3^, Xiying Chen^1^, Qiaodan Hu^3^, Haimei He^3^, Jiawei Wu^3^, Leyan Shan^3^, Shihui Feng^3^, Yingzhi Li^1^, Xiongchang Tan^3^, Hao Wang^2,^ *, Zilong Wang^3, 4, 5,^ *, Chaoran Wu^1,^ *

^1^ Department of Anesthesiology, Shenzhen People’s Hospital, The Second Clinical Medical College, Jinan University, Shenzhen, Guangdong, China

^2^ Department of Anesthesiology and Clinical Research Institute, The First Affiliated Hospital, Shantou Clinical Medical College, Jinan University, Guangzhou, China

^3^ Department of Medical Neuroscience, School of Medicine, Southern University of Science and Technology, Shenzhen, Guangdong, China

^4^ Key University Laboratory of Metabolism and Health of Guangdong School of Medicine, Southern University of Science and Technology, Shenzhen, Guangdong, China

^5^ SUSTech Homeostatic Medicine Institute, School of Medicine, Southern University of Science and Technology, Shenzhen, Guangdong, China

***** Corresponding author: Chaoran Wu ([wu.chaoran@szhospital.com](mailto:wu.chaoran@szhospital.com)), Zilong Wang ([wangzl6@sustech.edu.cn](mailto:wangzl6@sustech.edu.cn)), Hao Wang ([haowang@jnu.edu.cn](mailto:haowang@jnu.edu.cn))

**Figure S1. Peripheral CB_1_R activation alleviates incision-induced postoperative pain.** (A, B) Intraplantar (i.pl.) injection of WIN 55,212-2 (WIN) suppresses incision-evoked mechanical allodynia on postoperative day 1 in both male and female WT mice. Two-way ANOVA with Sidak correction for multiple comparisons. ***P* < 0.01 and *****P* < 0.0001. (C) Systemic administration or contralateral (non-incised) i.pl. injection of 3 μg WIN cannot suppress incision-evoked mechanical allodynia on postoperative day 1 in WT mice. Two-way ANOVA with Sidak correction for multiple comparisons. ***P* < 0.01 and *****P* < 0.0001. (D) Representative mouse footfall pattern diagram illustrating stance and swing phases in gait analysis test. Solid bars denote stance phase; blank spaces denote swing phase. (E) Representative mouse plantar contact area-time graph in gait analysis test. (F-I) Statistical analysis of stance time, max contact area, print area, and print length for the mice incised right hind paw in gait analysis test. Two-way ANOVA with Sidak correction for multiple comparisons; 9 mice/group. (J, K) Schematic of the rotarod test and statistical results at different time points following i.pl. injection of WIN. Two-way ANOVA with Sidak correction for multiple comparisons; n = 9 mice/group. (L, M) Representative movement trajectories and statistical analysis of total distance traveled in the open field test during the 1 h observation period following i.pl. WIN administration in mice. Unpaired t tests; n = 7-8 mice/group. (N) i.pl. injection of WIN suppresses incision-evoked mechanical allodynia on postoperative day 1 in CB_2_R KO mice. (O) i.pl. injection of AM1241 doesn’t suppress incision-evoked mechanical allodynia on postoperative day 1 in WT mice. Two-way ANOVA with Sidak correction for multiple comparisons. ***P* < 0.01 and *****P* < 0.0001; sample sizes are indicated in parentheses. Means ± SEM. M, male; F, female; BL, baseline; s.c., subcutaneous; ips., ipsilateral; con., contralateral; LF, left forefoot; LH, left hindfoot; RF, right forefoot; RH, right hindfoot.

**Figure S2. Conditional knockout or antagonism of peripheral CB_1_R leads to significant** **mechanical allodynia.** (A, B) Genotype identification representative images of *Adv*;*Cnr1*^fl/fl^ mice and *Trpv1*;*Cnr1*^fl/fl^ mice. (C) Conditionally knockout *Cnr1* in all sensory neurons leads to significant mechanical allodynia in *Adv*;*Cnr1*^fl/fl^ mice post-incision. (D) Conditionally knockout *Cnr1* in TRPV1^+^ sensory neurons leads to significant mechanical allodynia in *Trpv1*;*Cnr1*^fl/fl^ mice post-incision. (E) i.pl. injection of CB_1_R antagonist AM251 induced significant mechanical allodynia in WT mice. Two-way ANOVA with Sidak correction for multiple comparisons. ***P* < 0.01 and *****P* < 0.0001. Sample sizes are indicated in parentheses; means ± SEM. L, leader; M, male; F, female; BL, baseline.

**Figure S3. Conditional knockout of the peripheral CB_1_R does not affect thermal nociception in mice** (A-C) Thermal nociception assessed by the tail-flick test in *Adv*;*Cnr1*^fl/fl^ mice and *Trpv1;Cnr1*^fl/fl^ mice. (D-F) Thermal nociception assessed by hot-plate test in *Adv*;*Cnr1*^fl/fl^ mice and *Trpv1;Cnr1*^fl/fl^ mice. Unpaired t tests; n = 5-10 mice/group; means ± SEM.

**Figure S4.** **Conditional knockout of *Cnr1* in sensory neurons results in increased expression of ASIC3 in DRG.** (A, B) Basal ASIC3 protein levels in the DRG of WT mice and *Adv;Cnr1*^fl/fl^ mice. Unpaired t tests; n = 5 mice/group. (C, D) Basal ASIC3 protein levels in the DRG of WT mice and *Trpv1;Cnr1*^fl/fl^ mice. Unpaired t tests; n = 5 mice/group; means ± SEM.

**Figure S5. The activation of CB_1_R inhibits ASIC3-mediated nociceptive behavior.** (A) i.pl. GMQ-induced licking behaviors in male and female WT mice. Two-way ANOVA with Sidak correction for multiple comparisons; n = 5-11 mice/group. (B) i.pl. GMQ-induced licking behaviors decreased in ASIC3 KO mice (left: time course; right: total licking duration). Unpaired t tests; n = 8 mice/group. (C) i.pl. GMQ-induced mechanical allodynia abolished in ASIC3 KO mice. Two-way ANOVA with Sidak correction for multiple comparisons, **P* < 0.05, ***P* < 0.01, and *****P* < 0.0001; n = 8 mice/group. (D) Effect of co-administration of CB_1_R antagonist AM251 on WIN-mediated inhibition of GMQ-induced licking behaviors (left: time course; right: total licking duration). One-way ANOVA with Tukey's multiple comparisons test, **P* < 0.05. (E) The inhibitory effect of 15 min pretreatment with WIN (i.pl.) on GMQ-induced mechanical allodynia in WT mice. Two-way ANOVA with Sidak correction for multiple comparisons, **P* < 0.05. Sample sizes are indicated in parentheses; means ± SEM. M, male; F, female; BL, baseline.

**Figure S6. CB_1_R and ASIC3 are co-expressed in the CGRP^+^ DRG neurons.** (A) UMAP plot revealing that the *Asic3* and *Cnr1* genes are expressed mainly in the *Calca^+^* DRG neuron subpopulations of mice. (B) Size distribution patterns of ASIC3^+^, *Cnr1*^+^, CGRP^+^ neurons, and total neurons in the DRGs. n = 5 mice/group, 3 slides/mouse. (C) UMAP plot revealing the gene expression profiles of *Asic1* and *Asic2* in cells of the mouse spinal cord.

**Figure S7. APETx2 inhibits GMQ-induced ASIC3 activation.** (A-C) Representative traces and quantifications of pretreatment with 1 μM APETx2 (2 min) to 3 mM GMQ (10 s)-induced inward currents in CHO cells. Paired/unpaired t tests; n = 13-14 cells/groups. (D, E) Representative action potential traces and quantification of firing frequency in cultured WT DRG neurons from naïve mice or incised mice. Two-way ANOVA with Sidak correction for multiple comparisons; n = 12-13 cells/group from 3 mice. (F, G) Representative traces and quantifications of rheobase of cultured WT DRG neurons from naïve mice or incised mice. (H) Quantifications of resting membrane potentials (RMP) of cultured WT DRG neurons from naïve mice or incised mice. Unpaired t tests; n = 12-13 cells/group from 3 mice. (I, J) Representative action potential traces and quantification of firing frequency in cultured WT DRG neurons following treatment with GMQ (3 mM) alone or in combination with APETx2 (1 μM). Two-way ANOVA with Sidak correction for multiple comparisons; n = 12-13 cells/group from 3 mice. (K, L) Representative traces and quantifications of rheobase of cultured WT DRG neurons after APETx2. (M) Quantifications of resting membrane potentials (RMP) of cultured WT DRG neurons after APETx2 treatments. Unpaired t tests; n = 12-13 cells/group from 3 mice. Means ± SEM.

**Figure S8. ASIC3 blocker inhibits *Cnr1* knockout-induced hyperexcitability of sensory neurons.** (A, B) Representative traces and quantifications of action potentials of primary cultured DRG neurons from *Trpv1*;*Cnr1*^fl/fl^ mice. Two-way ANOVA with Sidak correction for multiple comparisons; n = 10-12 cells/group from 3 mice. (C, D) Representative traces and quantifications of rheobase of primary cultured DRG neurons from *Trpv1*;*Cnr1*^fl/fl^ mice. (E) Quantifications of resting membrane potentials (RMP) of primary cultured DRG neurons from *Trpv1*;*Cnr1*^fl/fl^ mice. One-way ANOVA with Tukey's multiple comparisons test; n = 10-12 cells/group from 3 mice; means ± SEM.

**Figure S9. Knockout ASIC3 inhibits the phosphorylation of ERK_1/2_ in DRG and promotes the resolution of postoperative pain in rats.** (A, B) pERK_1/2_ levels in DRGs from WT and ASIC3 KO rats on postoperative day 1. One-way ANOVA with Tukey's multiple comparisons test; n = 8 rats/group. (C) The inhibitory effect of 15 min pretreatment with ERK inhibitor PD98059 (i.pl.) on GMQ-induced mechanical allodynia in WT mice. Two-way ANOVA with Sidak correction for multiple comparisons, **P* < 0.05; n = 9-10 mice/group. (D) Mechanical paw withdrawal threshold in wild-type (WT) and ASIC3 KO rats following plantar incision. Two-way ANOVA with Sidak multiple comparisons, **P* < 0.05 and *****P* < 0.0001, n = 10-12 rats/group; means ± SEM. M, male; F, female; BL, baseline.
